# METTL3 suppresses neuropathic pain via modulating N6-methyladenosine-dependent primary miR-150 processing

**DOI:** 10.1038/s41420-022-00880-2

**Published:** 2022-02-24

**Authors:** Le Zhang, Xin Zhao, Jing Wang, Yanwu Jin, Moxuan Gong, Yuyang Ye, Peilong Li

**Affiliations:** 1 Department of Anesthesiology, The Second Hospital, Cheeloo College of Medicine, Shandong University, Jinan, 250033 Shandong Province China; 2 Department of Clinical Laboratory, The Second Hospital, Cheeloo College of Medicine, Shandong University, Jinan, 250033 Shandong Province China

**Keywords:** Neuropathic pain, Mechanisms of disease

## Abstract

Methyltransferase-like 3 (METTL3)-modulated N6-methyladenosine (m6A) was recently identified as an important epigenetic regulation type during RNA processing and contributes to multiple pathological processes. Neuropathic pain (NP) is induced by a lesion of the somatosensory nervous system, and the detailed pathways by which METTL3/m6A regulated to modulate gene dysregulation and enable NP have remained unclear. Therefore, this study investigated the function of METTL3-mediated m6A methylation on miRNA maturation, and investigated how this regulation contributes to NP progression. A rat model characterized with typical NP was established by a spared nerve-injury (SNI) method. By analyzing the expression levels of METTL3 and m6A methylation, we found that METTL3, along with m6A methylation, was dramatically downregulated in NP rats in contrast to the sham ones. Functionally, enhanced METTL3 promoted the m6A methylation in total RNAs and inhibited NP progression, whereas silencing METTL3 suppressed m6A methylation and increased NP severity. Mechanistically, METTL3 accelerated miR-150 maturation via mediating m6A methylation of primiR-150 at locus 498, cooperating with the “m6A reader” YTHDF2. Meanwhile, miR-150 could directly target brain-derived neurotrophic factor (BDNF) mRNA, and the METTL3/miR-150/BDNF regulatory pathway was finally established. Clinically, we proved that serum METTL3 mRNA was also downregulated in Shingles patients with NP, suggesting its diagnostic potential. In conclusion, we demonstrated an essential function of METTL3-regulated N6-methyladenosine during NP progression via modulating primiR-150 maturation. Serum METTL3 could effectively differentiate NP patients from healthy people, and is useful for dynamic monitoring of diseases after treatment. Therefore, the METTL3/miR-150/BDNF pathway may be a promising therapeutic target for NP patients.

## Introduction

Neuropathic pain (NP) is a pain disorder caused directly by lesions or tissue damage affecting the somatosensory system [1]. Patients with NP usually showed clinical symptoms closely associated with hyperalgesia, which could be caused by several types of diseases, such as Shingles [2]. Another characteristic of NP in clinical progression is often accompanied with spinal or peripheral cord injury, which could be found during orthopedics-related diseases [3]. Due to the complex causes and disease process, the theoretical basis for NP progression is largely unknown. Thus, uncovering the precise pathogenesis of NP will help provide useful therapeutic targets and clinical diagnostic markers.

Recently, the emerging of N6-methyladenosine (m6A) has attracted more and more attention due to its broad role in various biological functions, especially during the RNA synthesis, maturation, and translational regulations [4]. A series of proteins were tightly associated with the m6A modifications, including the “erasers” fat mass and obesity-associated protein (FTO) and AlkB homolog H5 (ALKBH5), the “writers” methyltransferase-like 3 (METTL3), methyltransferase-like 14 (METTL14), and Wilms’ tumor 1-associated protein (WTAP), and the “readers” hnRNPA2B1 and IGF2BP1/2 [5]. Dysregulations of m6A modifications could influence extensive biochemical processes, causing regulatory imbalance and development of clinical diseases [6]. Until now, the emerging functional association between m6A methylation and NP is still not well known and new researches are urgently needed.

Importantly, a series of studies demonstrated that m6A methylation played essential roles in RNA biogenesis at the posttranscriptional level, for example, Frye et al. revealed that m6A was involved in the RNA-stability regulation of the modified transcripts, indicating a potential regulatory pathway to coordinate the processing of transcripts during cell homeostasis and molecular translocations [7]. Another study reported by Alarcón et al. revealed that the METTL3-mediated m6A modification increased the connection of DGCR8 to pri-microRNAs, facilitating the formation process of miRNA maturation and balance disorders [8]. Therefore, m6A-mediated microRNA processing may be critical for NP progression.

In our study, we sought to define the functional role of METTL3 in NP progression and elucidate the regulatory pathway by which METTL3 regulates miRNA maturation to participate in NP. Our preliminary data indicated that METTL3 was downregulated in lumbar spinal cord of SNI-NP rat and promoted NP progression. Then, miR-150 was verified as a targeted transcript of METTL3. Moreover, deletion of METTL3 suppressed miR-150 level by mediating DGCR8 binding with miR-150 in an m6A-dependent manner. Finally, we proved that METTL3 inhibited NP progression through targeting the miR-150/BDNF signaling pathway.

## Results

### METTL3, along with m6A methylation, was downregulated in NP rats undergoing SNI

**Fig. 1:**
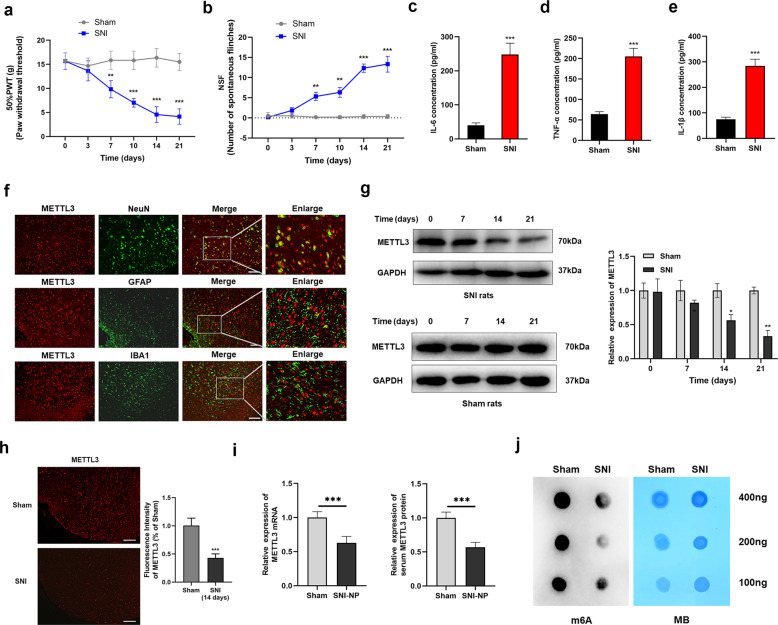
METTL3 was lowly expressed in the spinal cord of established SNI-NP rats. **a**, **b** PWT (**a**) and NSF (**b**) were evaluated in the established SNI model rats and controlled sham ones. *N* = 5, **P < 0.01, ***P < 0.001 compared with sham groups. **c–e** Neuroinflammatory factors, including IL-6 (**c**), TNF-α (**d**), and IL-1β (**e**) were upregulated in spinal cord tissues of SNI-NP rats in contrast to sham ones. *N* = 3, ***P < 0.001 compared with sham groups. **f** Double-fluorescent labeling experiment was performed with the respective antibodies to verify the tissue location of METTL3. The indications of green markers were as follows: NeuN indicates neuron, lba1 indicates microglia and GFAP indicates astrocyte. Scale bar = 100 μm. **g**, **h** METTL3 protein was detected in experimental NP rats and sham control ones according to the results obtained from western blot (**g**) and immunofluorescence (**h**) at the respective time points. Scale bar = 100 μm. *P < 0.05, **P < 0.01, ***P < 0.001 compared with sham rats. **i** The transcript and protein levels of METTL3 were detected in serum of SNI-NP rats and sham rats via qPCR and ELISA (repeated triplicates). ***P < 0.001. **j** m6A dot-blot assays of spinal cord tissues from SNI and sham rats. Methylene blue (MB) stain as loading control. The results showed a decreased methylation of RNA in SNI rats compared with sham ones. The experiment was repeated three times with similar results. Data are presented as mean ± SD.

To determine the expression of METTL3, we constructed the SNI NP rat model. As shown, no significant difference of PWT and NSF between sham and experimental groups was identified before the experiment started. As shown, a decreased PWT and an elevated NSF were verified in NP rats, however, this change was not observed in the sham group (Fig. 1a and b). In addition, we determined the levels of IL-6, TNF-α, and IL-1β, and found that these cytokines were significantly upregulated in SNI-NP rats according to ELISA results (Fig. 1c–e). Then, we performed immunofluorescence to verify the location of METTL3 in the spinal cord. As shown, METTL3 mainly coexisted with NeuN (a specific neuronal marker) and very few METTL3-detectable cells were colocated with glial fibrillary acidic protein (GFAP) (a specific astrocyte marker) or IBA1 (a specific microglial marker), suggesting that METTL3 is predominantly expressed in neurons of the spinal cord (Fig. 1f). By detecting the expression of METTL3 using lumbar spinal cord samples from SNI-NP rats and sham ones, we found that METTL3 was significantly downregulated in NP rats at both transcript and protein levels (Fig. 1g and h). Meanwhile, we found that METTL3 expression was also decreased in serum samples of NP rats compared with sham rats according to qRT-PCR and ELISA results (Fig. 1i). Since METTL3 is closely associated with m6A methylation, we evaluated the m6A enrichment in the spinal cord and revealed that m6A level was dramatically suppressed after SNI surgery when compared with sham groups on day 14 in the L3/5 spinal cord (Fig. 1j), suggesting a consistent trend with spinal METTL3. Collectively, our data provided clear evidence that supports a suppressed expression of METTL3 during NP progression.

### Downregulation of METTL3 is required for reduction of m6A and related to NP progression

**Fig. 2:**
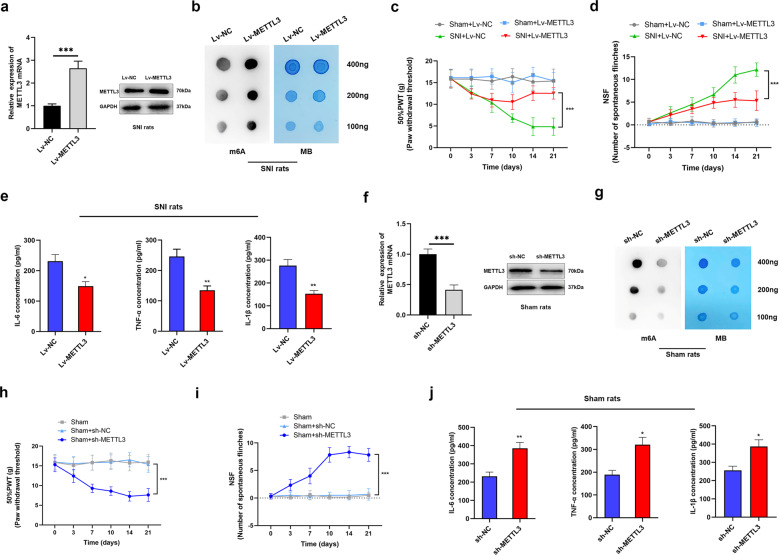
METTL3 plays a suppressive role in NP progression. **a** METTL3 was overexpressed in spinal cord tissues of SNI rats after injection of Lv-METTL3 vectors. ***P < 0.001. **b** m6A dot blot assays of spinal cord tissues after injection of METTL3 overexpression vectors into SNI rats. Methylene blue (MB) was used as experimental controls. The results showed an increased methylation of RNA upon overexpression of METTL3. **c**, **d** PWT (**c**) and NSF (**d**) analyses revealed that METTL3 overexpression significantly reversed the NP status of SNI rats, but showed no effect on sham rats. *N* = 5 biological replicates. ***P < 0.001. **e** IL-6, TNF-α and IL-1β were detected in SNI rats via ELISA-overexpressed METTL3, and a significantly decreased expression of the above markers were identified. *N* = 3 biological replicates. *P < 0.05, **P < 0.01 compared with Lv-NC group. **f** The expression of METTL3 was detected after injection of METTL3-silencing vectors into experimental rats, and the results showed that METTL3 was significantly downregulated. *N* = 3 biological replicates. ***P < 0.001. **g** Dot-blot assays with m6A antibody in spinal cord tissues after injection of METTL3-silencing lentivirus into SNI rats. Methylene blue (MB) was set as the loading control. A dramatically decreased methylation of RNA was found when METTL3 was silenced. **h**, **i** PWT (**h**) and NSF (**i**) of sham rats in the respective groups were tested. Silence of METTL3 induced the NP in sham rats. *N* = 5 biological replicates. **P < 0.01. **j** Inflammatory markers IL-6, TNF-α, and IL-1β were detected via ELISA and found to be upregulated by the silence of METTL3 in sham rats. *P < 0.05, **P < 0.01 compared with control group. The experiment was repeated three times with similar results.

To define the essential function of METTL3 in NP progression, we overexpressed METTL3 by intrathecal administration of LentiMettl3 (Lv-METTL3). The data showed that Lv-METTL3 reversed the decreased levels of METTL3 transcript and protein (Fig. 2a). Meanwhile, the overexpression of METTL3 elevated m6A level when compared with an empty vector (Lv-NC) in the L3/5 spinal cord by intrathecal injection in SNI rats (Fig. 2b). In addition, overexpressed METTL3 significantly inhibited NP behaviors as evidenced by the elevated PWT and suppressed NSF in SNI rats, however, this influence was not observed in sham group (Fig. 2c and d). Consistently, expression levels of IL-6, TNF-α, and IL-1β were also suppressed by the overexpressed METTL3 (Fig. 2e), suggesting that enhanced METTL3 restrained the level of neuroinflammation in SNI rats.

On the other hand, we silenced METTL3 by constructing the respective lentivirus-shRNA in sham rats. The levels of spinal METTL3 mRNA and protein were silenced by the intrathecal injection of METTL3-silencing vector in sham rats (Fig. 2f), and the amount of m6A in the spinal cord was consistently, downregulated accordingly after intrathecal injection of sh-METTL3 (Fig. 2g). As shown, we found that knockdown of METTL3 induced NP in sham rats with decreased PWL and elevated NSF (Fig. 2h and i), and upregulated IL-6, IL-1β, and TNF-α levels (Fig. 2j). These results proved our hypothesis that METTL3 was essential for the modulation of spinal m6A in neuroinflammation of NP behavior model, and m6A methylation may be critical for the formation of chronic NP.

### METTL3-mediated methylation facilitates miR-150 processing via DGCR8

**Fig. 3:**
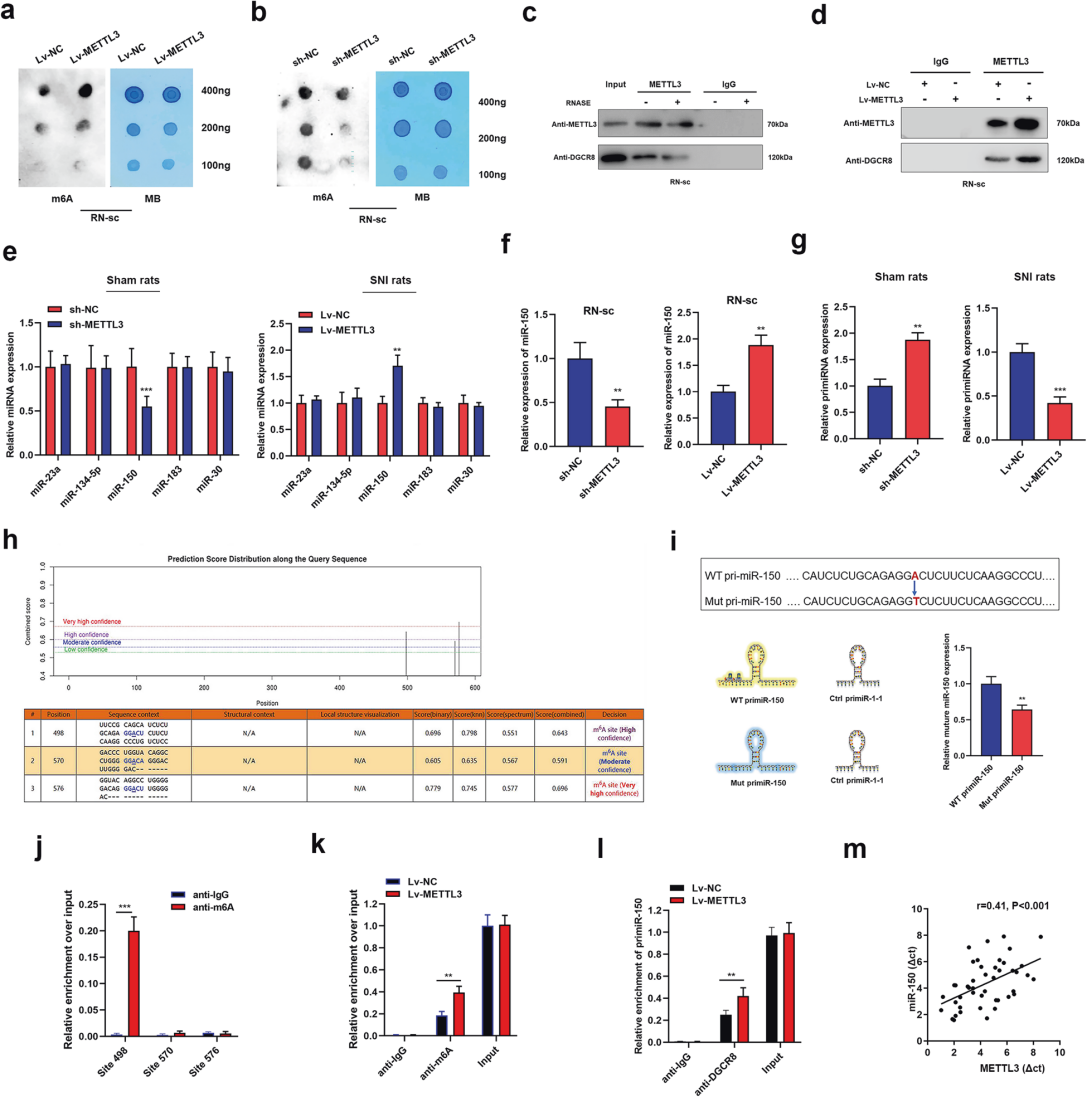
METTL3 facilitates miR-150 maturation via binding with DGCR8. **a**, **b** m6A dot blot assays of m6A content in RN-sc cells that were overexpressed (**a**) or silenced with METTL3 (**b**). Results showed a clear positive regulation of METTL3 on m6A enrichment. **c** Co-immunoprecipitation assay was performed using indicated antibodies. The results showed that METTL3 was directly connected with DGCR8 protein. **d** Immunoprecipitation assay was conducted with antibodies against DGCR8 and METTL3. The RNA pulldown from METTL3 overexpressed cells and NC cells was shown. **e** The expression of putative miRNAs in spinal cord tissues upon dysregulation of METTL3. The obtained data showed that miR-150 was silenced by METTL3 knockdown in sham rats, whereas upregulated when METTL3 was overexpressed in SNI rats. **P < 0.01, ***P < 0.001 in comparison with control groups. **f** miR-150 was detected in RN-sc cells upon silence or overexpression of METTL3. **P < 0.01 compared with control groups. **g** PrimiR-150 was quantified via qPCR analysis in spinal cord tissues injected with sh-METTL3 (sham) and Lv-METTL3 (SNI). **P < 0.01, ***P < 0.001 compared to control groups. **h** Potential targeted m6A sites in primiR-150 according to SRAMP online website. **i** Representation of the reporters constructed to confirm the functional role of METTL3 in miR-150 maturation. **j** Me-RIP assay showed a significant higher enrichment of m6A at site 498 compared with IgG control. *N* = 3 biological replicates. ***P < 0.001. **k**, **l** RIP assay with m6A antibody (**k**) and DGCR8 antibody (**l**) showed that the enrichment was increased upon METTL3 overexpression. *N* = 3 biological replicates. **P < 0.01. **m** qPCR showed a positive correlation between METTL3 transcript and miR-150 in serum of 45 NP patients. The experiment was repeated three times with similar results.

Recently, a study conducted by Alarcon R.’s lab revealed that METTL3 participated in miRNA processing and maturation via binding with DGCR8 protein [8]. Thus, we suppose that METTL3 may regulate NP progression via participating in the maturation of miRNAs by DGCR8. We first tested the m6A methylation level after overexpression of METTL3 in RN-sc cells. As shown, m6A methylation was significantly increased in RN-sc cells upon transfection of Lv-METTL3, whereas downregulated by the deletion of METTL3 (Fig. 3a and b). Next, by performing co-immunoprecipitation, we observed a direct interaction between METTL3 and DGCR8, and this interaction could be reduced by Rnase (Fig. 3c). We also observed that the interaction between DGCR8 and miRNAs was higher after METTL3 was overexpressed in RN-sc cells (Fig. 3d), indicating that the interaction between miRNAs and DGCR8might be modulated by METTL3-induced m6A enrichment.

Previous studies revealed that a series of miRNAs were involved in NP progression, including miR-23a [9], miR-150 [10, 11], miR-134-5p [12], miR-183 [13], and miR-30 [14]. Interestingly, only miR-150 was significantly elevated upon upregulation of METTL3 in SNI rats and decreased by METTL3 deletion in sham rats (Fig. 3e). In addition, this regulation of miR-150 was further confirmed in neuron cells (Fig. 3f). Moreover, an accumulation of primiR-150 was identified in METTL3-silenced sham rats and a decreased primiR-150 was found in METTL3-overexpressed SNI rats (Fig. 3g). To validate that m6A methylation modulated by METTL3 plays essential roles in miR-150 processing, we analyzed the potential m6A targeting motifs using at SRAMP (http://www.cuilab.cn/sramp). A total of 3 RRACH m6A-binding motifs situated in primiR-150 were identified (Fig. 3h), among which adenosine [A] at site 498 was not located in the pre-miRNA sequence (Supplementary Fig. S2). Thus, this m6A motif was mutated and this mutation dramatically suppressed the maturation process of miR-150 (Fig. 3i). MeRIP assay showed that m6A was enriched at locus site 498, and enhanced METTL3 increased the m6A methylation level of primiR-150 (Fig. 3j and k). In addition, the interaction between DGCR8 and primiR-150 was also promoted by METTL3 overexpression (Fig. 3l). By detecting the expression of METTL3 and miR-150 in serum samples of NP patients, we revealed a positive correlation between METTL3 and miR-150 (Fig. 3m). Altogether, our results suggested that METTL3 induced miR-150 processing by mediating DGCR8 recognition in an m6A-dependent manner.

### YTHDF2 is essential for the METTL3-mediated m6A modification of primiR-150

**Fig. 4:**
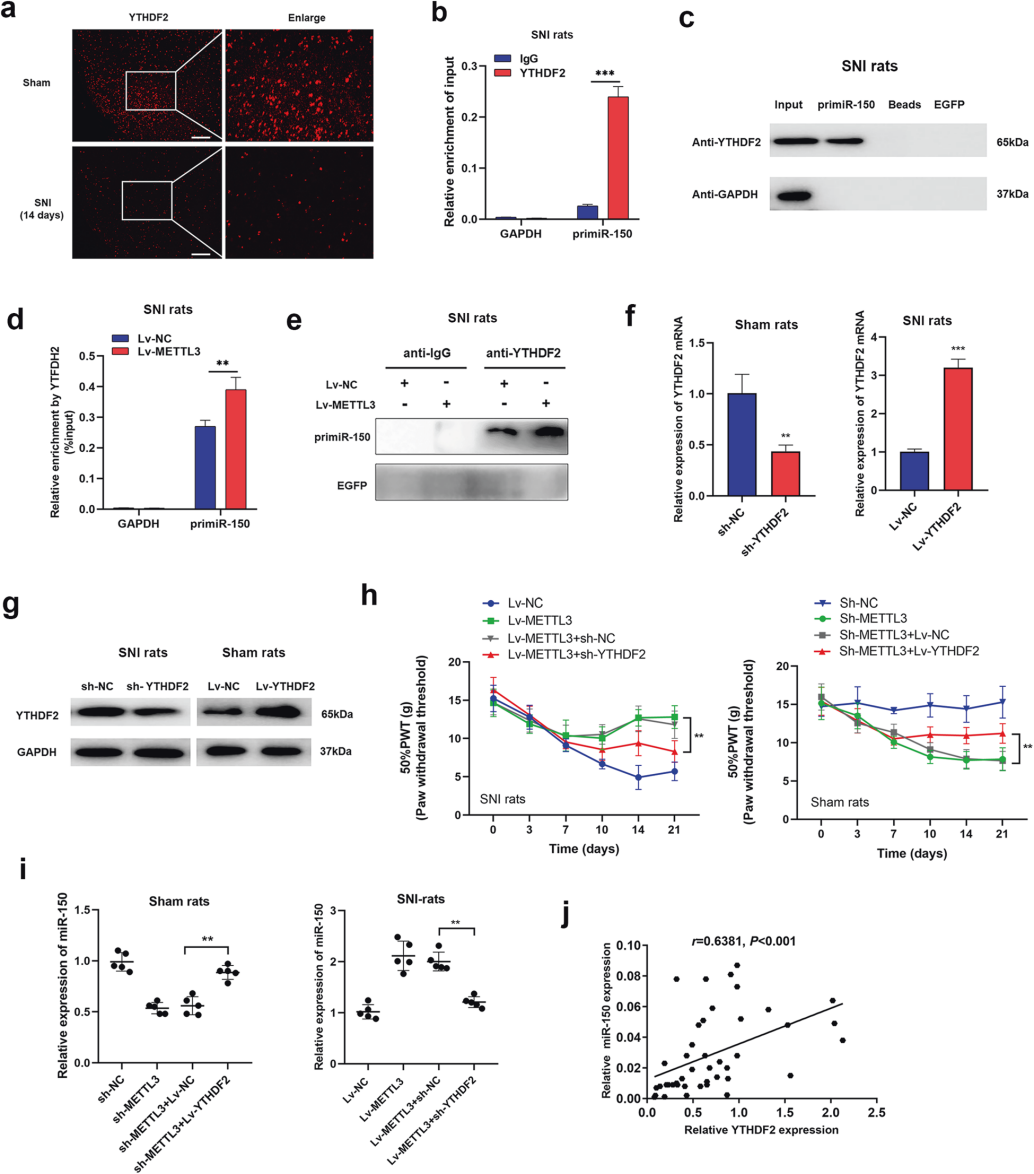
YTHDF2 cooperating with METTL3 regulates m6A methylation of primiR-150. **a** Immunofluorescence assay of YTHDF2 expression in spinal cord tissues from SNI rats at day 14 after model establishment, and red signals showed the positive staining sites. Scale bar = 100 μm. **b** RIP assay showed an increased binding of primiR-150 by YTHDF2, GAPDH mRNA was set as a nontarget control. ***P < 0.001. **c** RNA pulldown assay proved that the YTHDF2 protein was directly associated with primiR-150, EGFP RNA was used as RNA control. **d** RIP assay of the enrichment of primiR-150 by YTHDF2 in SNI rats overexpressed METTL3, and an increased enrichment caused by METTL3 was obtained, GAPDH mRNA was used as a nontarget control. **P < 0.01. **e** RNA-pulldown assay was performed in SNI rat overexpressed METTL3, and the YTHDF2 pull down by primiR-150 was significantly elevated upon upregulation of METTL3, EGFP RNA was set as RNA control. **f**, **g** Verification of the manipulations of YTHDF2 in SNI or sham rats at transcript (**f**) and protein levels (**g**). *N* = 3, **P < 0.01, ***P < 0.001 in comparison with the control group. **h** PWT of rats in the respective groups was performed when METTL3 and YTHDF2 were co-expressed. The results proved that YTHDF2 could effectively reverse the effects on PWT caused by METTL3. *N* = 5 biological replicates. **P < 0.01 compared with the control group. **i** Mature miR-150 expression was detected via qRT-PCR in sham and SNI rats after injection of METTL3 and YTHDF2. YTHDF2 could effectively reverse the effects on miR-150 expression mediated by METTL3. *N* = 5 biological replicates. **P < 0.01. **j** YTHDF2 and mature miR-150 levels were detected in 45 clinical NP patients and the two transcripts were positively expressed according to Spearman correlation test.

After revealing the essential role of METTL3-mediated m6A methylation of miR-150 in NP, we next explored the collaborated reader proteins. It is well known that YTH-domain family 2 (YTHDF2) was tightly associated with the m6A-related RNA degradation [15, 16], we make a hypothesis that YTHDF2 may collaborate with METTL3 to exert a promethylation role during miR-150 maturation. YTHDF2 was detected in spinal cord tissues of SNI and sham rats, and an obvious decreased expression was identified in SNI rats at day 14 according to immunofluorescence assay (Fig. 4a). According to our RIP data with spinal cord tissues from NP rats, primiR-150 was significantly enriched by YTHDF2 (Fig. 4b). This was further confirmed by performing RNA-pulldown assay with specific primiR-150 probe (Fig. 4c). Furthermore, enhanced METTL3 significantly increased the interaction between YTHDF2 and primiR-150 as shown in RIP and pulldown assays (Fig. 4d and e). Then, we silenced and overexpressed YTHDF2 expression in sham and SNI rats, respectively (Fig. 4f and g). As shown, silencing YTHDF2 almost reversed the METTL3-mediated NP relief in SNI rats, while overexpression of YTHDF2 abrogated the METTL3-induced NP progression in sham ones (Fig. 4h). Consistently, the suppression of miR-150 caused by sh-METTL3 was rescued by overexpression of YTHDF2 in sham rats; whereas METTL3-induced miR-150 accumulation was reversed by YTHDF2 silencing (Fig. 4i). Finally, the expression of YTHDF2 was positively correlated with miR-150 level in NP patients with Shingles (Fig. 4j), which further supported our conclusion. Taken together, these results revealed that YTHDF2, along with METTL3, mediated m6A methylation and regulated the processing of miR-150.

### miR-150 is a functional target of METTL3 for NP progression

**Fig. 5:**
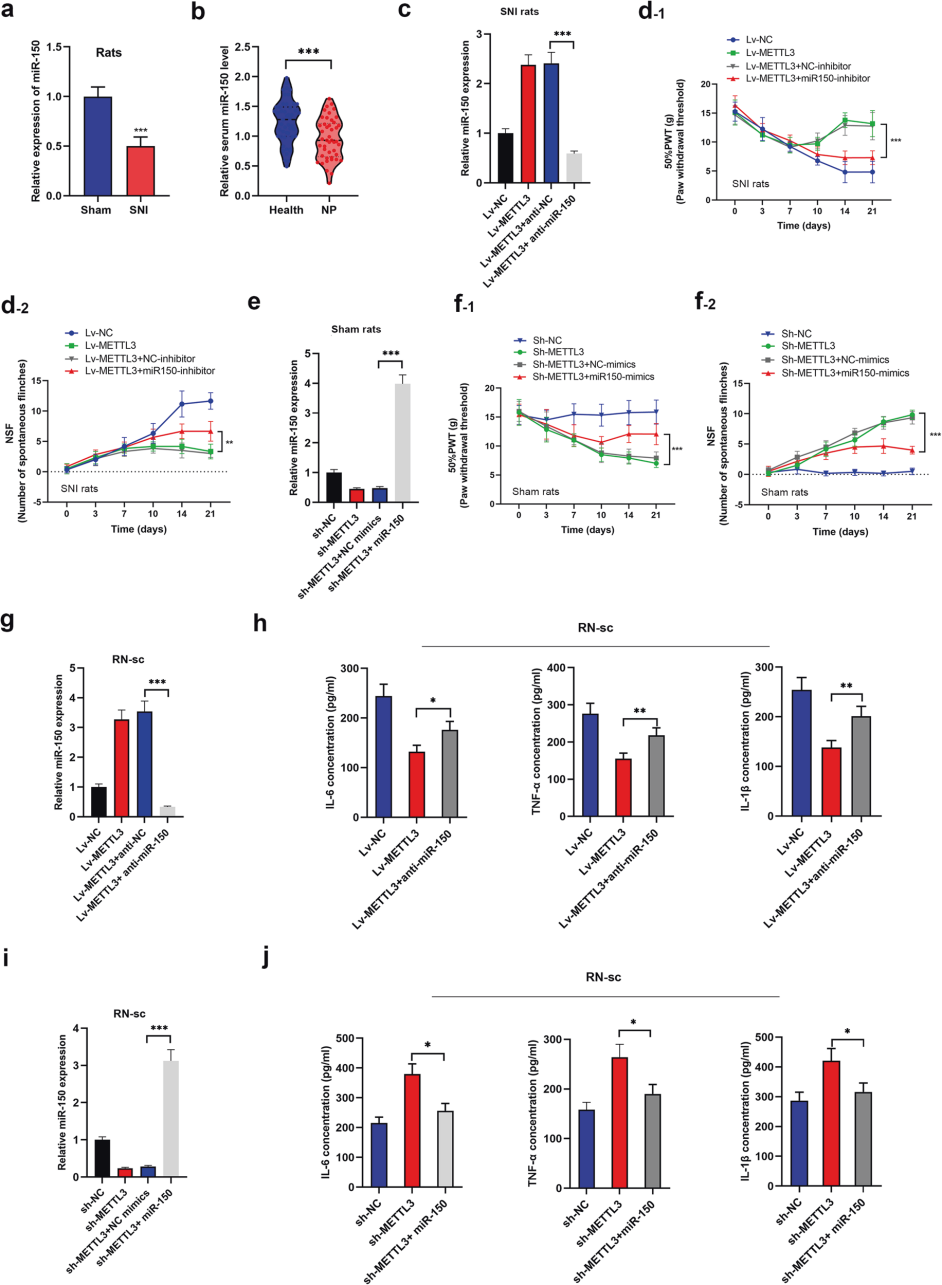
miR-150 was functionally targeted by METTL3. **a** miR-150 was detected in spinal cord tissues, and a significant upregulated miR-150 was observed in SNI rats compared with sham ones. ***P < 0.001 in comparison with sham group. **b** Serum miR-150 was also suppressed in 45 patients with Shingles. ***P < 0.001. **c** miR-150 was quantified via qPCR in the respective groups. A significant downregulation of miR-150 was observed when lentivirus–antimiR-150 was injected (repeated in triplicates). ***P < 0.001. **d** PWT (**d**_**-1**_) and NSF (**d**_**-2**_) were monitored in SNI-NP rats with METTL3 overexpression and miR-150 knockdown. Knockdown of miR-150 reversed the METTL3-mediated NP suppression. *N* = 5 biological replicates. ***P < 0.001. **e** miR-150 was determined in respective groups and overexpressed by injection of miR-150 mimics (repeated in triplicates). ***P < 0.001. **f** PWT (**f**_**-1**_) and NSF (**f**_**-2**_) were recorded in sham rats with METTL3 silencing and miR-150 overexpression. MiR-150 could significantly rescue the METTL3-induced NP behaviors (repeated five times). **P < 0.01. **g** miR-150 was manipulated in RN-sc cells from the respective groups (*N* = 3). ***P < 0.001. **h** IL-6, TNF-α and IL-1β were evaluated by ELISA in RN-sc cells of the respective groups, and the expression of the above inflammatory markers was rescued by silence of miR-150. *N* = 3 biological replicates. *P < 0.05, **P < 0.01. **i** miR-150 expression was validated upon silence of METTL3 and overexpression of miR-150 (repeated in triplicates). ***P < 0.001. **j** Influence of METTL3/miR-150 was evaluated on the expression of IL-6, TNF-α, and IL-1β by performing ELISA. Enhanced miR-150 significantly reversed METTL3-induced expression of those inflammatory markers (repeated in triplicates). *P < 0.05.

To define whether miR-150 is essential for METTL3-induced NP, we first detected the concentration of miR-150 in SNI-NP rat tissues and patient samples. Figure 5a shows that miR-150 was dramatically downregulated in NP rats in contrast to control ones. Consistently, miR-150 expression was also suppressed in clinical NP patients in contrast to healthy controls (Fig. 5b). Next, we injected miR-150 silencing lentivirus into METTL3-overexpressed SNI rat model and found that silence of miR-150 could partially reverse the METTL3-induced aberrant PWT and NSF (Fig. 5c and d). Meanwhile, overexpression of miR-150 could abrogate the sh-METTL3-mediated progression of NP in sham rats (Fig. 5e and f). We also performed in vitro restoration assays by transfection of miR-150 inhibitors in rat neuron cells (Fig. 5g). As expected, inhibition of miR-150 in RN-sc cells dramatically reversed the METTL3-mediated suppression of IL-6, TNF-α, and IL-1β (Fig. 5h); while miR-150 mimics could restore the sh-METTL3-caused upregulation of neuroinflammation markers (Fig. 5i and j), suggesting that miR-150 was essential for METTL3-modulated NP in vitro and in vivo.

### BDNF directly targeted by miR-150 and regulated by METTL3

**Fig. 6:**
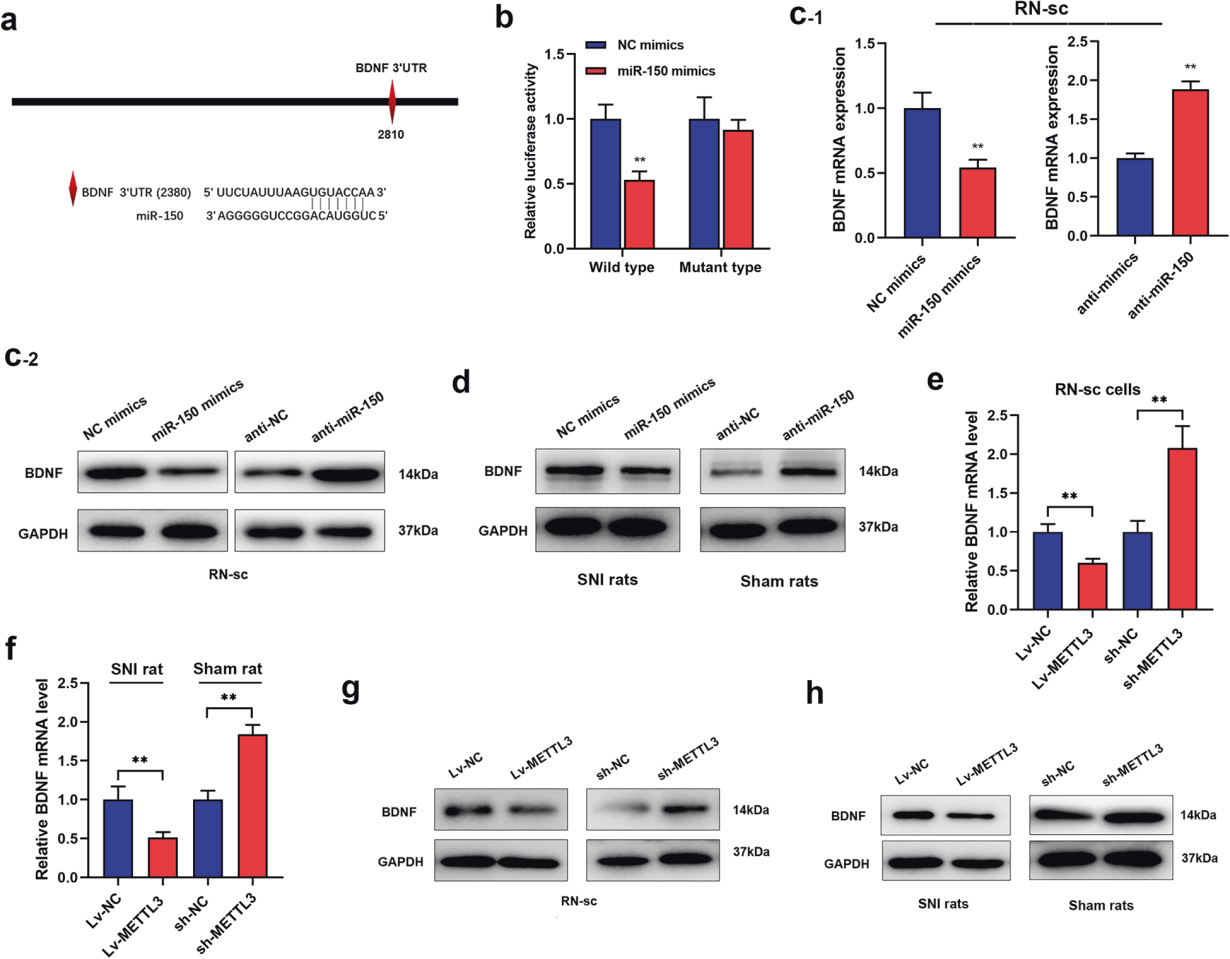
BDNF is a downstream target of METTL3/miR-150-regulatory axis. **a** Schematic representation of the putative miR-150-binding sites at the 3′-UTR area of BDNF. **b** Luciferase-reporter assay was performed and the mutant type of reporter abrogated the miR-150-induced signal inhibition (repeated in triplicates). **P < 0.01 in comparison with NC group. **c** BDNF mRNA (**c**_**-1**_) and protein (**c**_**-2**_) expressions were detected upon overexpression or silence of miR-150 (*N* = 3). **P < 0.01 in comparison with control group. **d** BDNF protein level in spinal cord tissues was evaluated via western blot upon overexpression of miR-150 in SNI rats and knockdown of miR-150 in sham rats. BDNF protein was negatively regulated in the above rat models. **e**, **f** BDNF mRNA was determined in RN-sc cells (**e**) and spinal cord tissues (**f**) from sham rats or SNI ones when METTL3 was dysregulated. BDNF transcript was negatively regulated by METTL3 expression (repeated in triplicates). **P < 0.01. **g**, **h** Effects of METTL3 on BDNF protein expression were evaluated via western blot analysis in RN-sc cells (**g**) and spinal cord tissues of SNI rats or sham ones (**h**). The experiment was repeated three times with similar results.

Our previous work revealed that brain-derived neurotrophic factor (BDNF) was upregulated in SNI-NP rat models and played critical roles in NP progression [17–19], we thus assume that BDNF may be involved in the NP regulated by METTL3/miR-150 axis. According to the miRNA target online software miRDB (http://mirdb.org/), there is a binding site of miR-150 at 3′-UTR area of BDNF (Fig. 6a). By generating and transfecting wild or mutant type of 3′-UTR sequence into plasmid reporter of BDNF in RN-sc cells, we conducted luciferase-reporter assay. As shown, cotransfection of miR-150 with wild type of BDNF reporter dramatically decreased the luciferase activity, while no significant change was found when using mutant type of plasmid reporter (Fig. 6b). Then, we detected whether BDNF was negatively regulated by miR-150. Interestingly, enhanced miR-150 decreased, whereas silence of miR-150 upregulated BDNF level in RN-sc cells. In addition, BDNF protein was also negatively regulated by miR-150 in SNI and sham rat models (Fig. 6d). As METTL3 and miR-150 were tightly interacted, we sought to define whether BDNF was also regulated by METTL3. As expected, we found that overexpression of METTL3 reduced, while deletion of METTL3 increased BDNF expression in RN-SC cells and SNI-NP rats (Fig. 6e and h). Collectively, our results verified that BDNF was directly targeted by miR-150 and inhibited by METTL3.

### METTL3 inhibits NP progression via the miR-150/BDNF signaling pathway

**Fig. 7:**
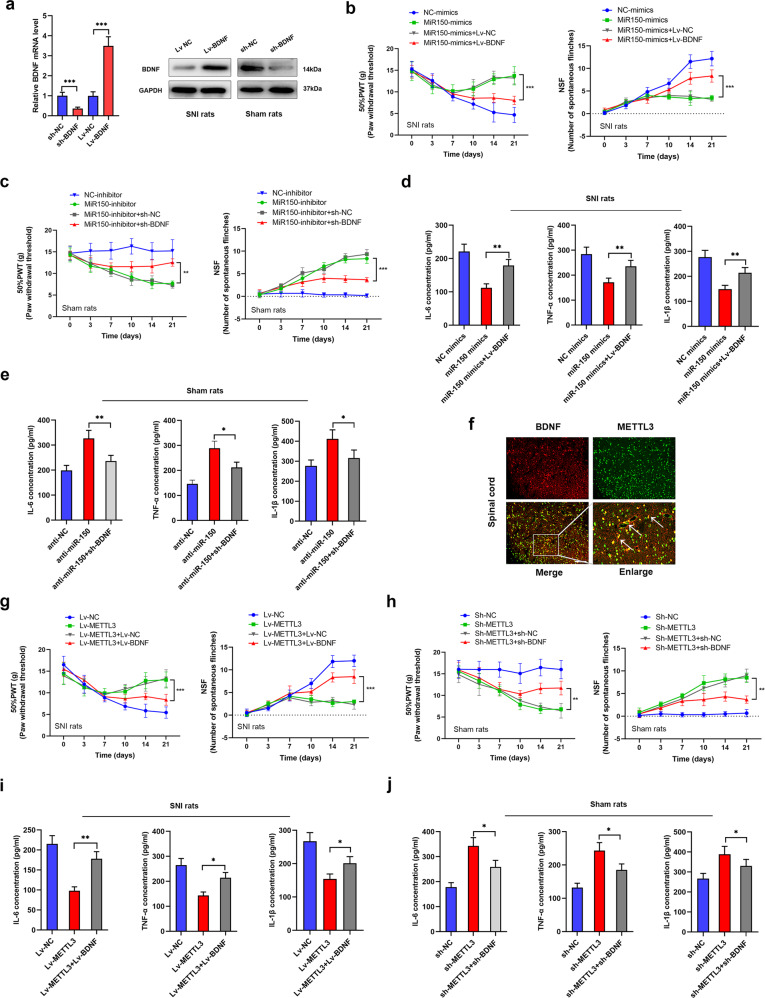
METTL3/miR-150 regulates NP through functionally targeting BDNF. **a** qPCR and western blot assay showed that BDNF was manipulated with the respective vectors at both mRNA and protein levels in SNI rats and sham rats (repeated in triplicates). ***P < 0.001. **b**, **c** PWT and NSF were recorded at the respective time points after overexpression of miR-150 and BDNF in SNI rats (**b**), and silencing of miR-150 and BDNF in sham rats (**c**). *N* = 5, ***P < 0.001. **d**, **e** Neuroinflammatory markers, including IL-6, TNF-α, and IL-1β, were evaluated via ELISA upon the respective manipulations. BDNF dysregulation could effectively restore the influence of miR-150 on inflammation (*n* = 3). *P < 0.05, **P < 0.01. **f** Co-immunofluorescence assays with BDNF and METTL3 antibodies showed that BDNF and METTL3 were co-localized in neurons of spinal cord area of SNI rats. Imagines were presented at a magnification of 200× and arrows indicate the representative locations. Scale bar = 100 μm. **g**, **h** METTL3- and BDNF-expressing vectors were generated and co-injected intrathecally into sham or SNI rats. The results showed that BDNF could dramatically reverse the METTL3-induced NP progression as evidenced by the respective PWT and NSF changes (*n* = 5). **P < 0.01, ***P < 0.001. **i**, **j** Dysregulation of BDNF could abrogate the METTL3-mediated effects on neuroinflammation according to the results of ELISA in SNI and sham rats (the experiment was repeated in triplicates). *P < 0.05, **P < 0.01.

To define that BDNF was a functional target of METTL3/miR-150 axis, and the regulatory mechanism of METTL3/miR-150/BDNF pathway in NP progression, we performed a series of restoration experiments. BDNF-overexpressing and -silencing lentivirus vectors were generated and intrathecally injected into sham and SNI rats, respectively (Fig. 7a). We found that miR-150 mimics elevated PWT and suppressed NSF in SNI rats, however, this effect was partly reversed by cotreatment with Lv-BDNF (Fig. 7b). Consistently, sh-BDNF abrogated the influence caused by miR-150 inhibitor in sham rats (Fig. 7c). Moreover, enhanced BDNF restored the miR-150-induced suppression of neuroinflammation (Fig. 7d), while silence of BDNF restrained miR-150-inhibitor-mediated upregulation of IL-6, TNF-α, and IL-1β (Fig. 7e), suggesting that BDNF may be essential for miR-150-mediated NP progression. To further verify the association between METTL3 and BDNF, we first performed co-immunofluorescence analysis. As shown, METTL3 and BDNF were mostly co-expressed in neurons of spinal cord tissues from SNI rats (Fig. 7f). Restoration experiments further showed that overexpression of BDNF in SNI rats could partly reverse METTL3-regulated suppression of NP progression in SNI rats (Fig. 7g), whereas deletion of BDNF interfered sh-METTL3-induced promotion of NP in sham rats (Fig. 7h). Consistently, dysregulated BDNF also reversed the METTL3-mediated regulation on neuroinflammation in both rat models (Fig. 7i and j). Taken together, we proved that BDNF is the functional target of METTL3/miR-150 signaling pathway.

### METTL3 could serve as a promising biomarker for NP patients

**Fig. 8:**
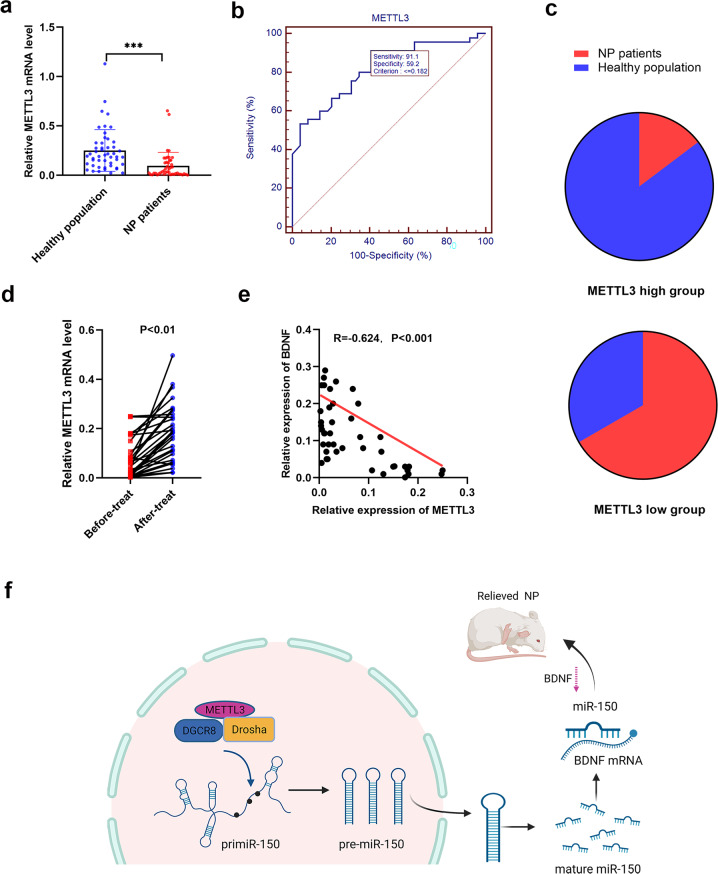
METTL3 is useful for diagnosis of clinical neuropathic pain. **a** METTL3 mRNA level was determined using qRT-PCR among patients with NP originated from Shingles. The results showed that METTL3 was dramatically downregulated in Shingles patients in contrast to controlled population (49 NP patients and 45 healthy people). ***P < 0.001. **b** The diagnostic value of METTL3 was investigated by establishing receiver operating characteristic curve based on the expression in serum samples. **c** The distribution analysis of the respective population revealed that patients with NP tend to express low level of METTL3. **d** METTL3 levels were monitored in patients before and after the respective therapy, and a significant upregulation of METTL3 was observed in 30 patients after treatment. **e** A significant negative correlation between serum METTL3 and BDNF levels was validated in 43 NP patients. **f** A summarized scheme showing the role of METTL3/miR-150/BDNF regulatory network in neuropathic pain. Data are presented as mean ± SD.

By using 45 serum samples from NP patients with Shingles, we investigated the clinical use of METTL3 for NP diagnosing. As shown, METTL3 level was downregulated in the patient group when compared with controlled nondisease population (Fig. 8a). Statistical analysis of ROC revealed that serum METTL3 provided a diagnostic sensitivity at 91.1%, specificity at 59.2%, and the area under the ROC curve was 0.817 (Fig. 8b). By using the stratification criterion obtained from the ROC curve (0.182), the total populations were grouped into a METTL3-high and a low-expressing population. Patient-distribution analysis suggested that the METTL3-high population consisted of much more percent of patients when compared with the METTL3-low population (Fig. 8c). Furthermore, after evaluating METTL3-expressing level in samples receiving effective therapy, we identified that METTL3 level was recovered compared with the level before treatment (Fig. 8d). In addition, METTL3 was significantly negatively correlated with BDNF level (Fig. 8e). Hence, our preliminary clinical investigations uncovered that circulating METTL3 could be useful for efficacy monitoring of clinical patients with neuropathic pain.

## Discussion

Neuropathic pain occurs after nerve injury, and harmful changes occur in the damaged neurons, which in turn affects the somatosensory system and downward regulatory pathways of the central nervous system [20, 21]. Understanding the molecular mechanism of NP is essential for developing effective therapeutic strategies. M6A-methylated noncoding RNAs tightly associated with the fate and functions of various biological processes, including carcinogenesis, cell growth, and neural signal transduction. Until now, whether m6A methylation participated in NP progression, if yes, the precise regulatory mechanism is not well defined. In this study, we focused on METTL3, one important member of so-called m6A writers, and revealed that METTL3 was significantly downregulated in SNI-NP rats and NP patients. Moreover, METTL3 suppressed NP through promoting the maturation process of miR-150 and the subsequent inhibition of BDNF expression (Fig. 8f). Our study demonstrated an essential role of METTL3-modulated m6A methylation in NP via binding with DGCR8 to accelerate miRNA synthesis.

Two major differences of pathological characteristics between neuropathic pain and non-neuropathic pain were identified. First, neuropathic pain is not transduced (noxious stimuli need to be converted into electrical impulses). Second, the prognosis of neuropathic pain is poor: damage to major nerves is more likely to cause chronic pain than damage to non-nerve tissues [22]. Clinically, NP more likely became tolerable to conventional analgesics such as nonsteroidal anti-inflammatory drugs and opioids than non-neuropathic pain [23]. The pathogenesis of NP is complicated, including changes in anatomical structure and functional impairment, and is often caused by multiple mechanisms, including peripheral sensitization, central sensitization, disability of the descending inhibitory system, activation of spinal cord glial cells, changes in ion channels. Recently, rat models have been widely used and accepted for NP investigation [24]. M6A methylation in RNAs has been widely reported and accepted as a critical regulation pathway during NP pathological processes [25]. One report by Li et al. revealed a critical role of “eraser” protein FTO-mediated m6A demethylase in NP progression [26], suggesting that m6A modification may also participate in NP. However, whether the methylation process modulated by METTL3/METTL14 “writers” contributes to NP is currently not reported yet.

It has been shown that the m6A methylation pattern could be reversed, during the process of involvement of methyltransferases (Writers), demethylases (Erasers), and methylated readers (Readers). Methyltransferases include METL3/14, WTAP, and KIAA1429, whose main role is to accelerate the m6A-binding progress. The demethylases, such as FTO and ALKHB5, are participating in demethylating sites that had been modified with m6A complex; while the role of Readers is to recognize bases that are methylated in progress, thereby activating downstream reactions with biological changes, including RNA maturation and stability control [27]. METTL3, along with METTL14, has been reported as the most widely regulatory m6A methyltransferase, and observed mostly located in nuclear speckles [28]. Recent studies clarified that METTL3 could participate in multiple diseases via regulating cancer cell proliferation [29], skeletal myoblasts [30], and embryonic development [31]. More recently, it is found that nervous system development and related disease progression were tightly associated with m6A RNA methylation [32]. Zhang et al. demonstrated that METTL3 induced chronic inflammatory pain in a mouse model of complete Freund’s adjuvant [33]. However, the role of METTL3 in NP is not clarified until now. Our study revealed a significant downregulation of METTL3 in the established SNI-NP rat model. Functionally, enhanced METTL3 suppressed NP in rats and RN-sc cells, uncovering an essential role in NP progression.

Recently, Alarcon et al. revealed that the METTL3-regulated m6A methylation could act on pri-miRNA and promote the process of mature miRNA formation via binding with microprocessor protein DGCR8. Subsequent studies further revealed that METTL3 could bind to DGCR8 and accelerate miRNA processing, including miR-375 [34], miR-218 [35], and miR-873 [36]. Those studies suggest that METTL3 may regulate pathological processes via the modulation of miRNA dysregulation and m6A methylation. In this report, we validated the expression of miRNAs, including miR-23a, miR-150, miR-134-5p, miR-183, and miR-30, and revealed that only miR-150 expression was controlled by METTL3. In addition, METTL3 modulated m6A methylation at the upstream site of pre-miR-150, which increased the processing of miR-150 through interacting with DGCR8 as evidenced by the MeRIP assay. MiR-150 was reported to participate in multiple biological processes, including neuropathic pain [37, 38]. Our study showed that miR-150 could suppress NP progression, and further reversed the sh-METTL3-mediated restoration of NP, which strengthened the role of miR-150 in NP and clarified the functional interaction between METTL3 and miR-150. In addition, YTHDF2 was verified to be essential for the process of primiR-150 into mature miR-150 by cooperation with METTL3.

It is well known that miRNAs are a group of noncoding RNAs with the length of 20–23 nucleotides, and commonly participated in the regulation of target genes at the posttranscriptional level [39]. Based on online prediction and experimental validation, we verified BDNF as the functional target of miR-150. BDNF is the most abundant neurotrophic factor in the body, and it works by combining with TrkB (tyrosine-kinase B), thereby activating the intracellular region, leading to enhanced autophosphorylation of TrkB, and activated Ras-MAPK pathway [40, 41]. Our study confirmed the role of METTL3/miR-150/BDNF signaling pathway in NP, providing strong evidence for the interaction between BDNF and NP. Finally, we revealed the clinical significance of serum METTL3 in patients with NP by serving as noninvasive diagnostic marker.

One limitation of this study is that the role of METTL3/miR-150/BDNF in clinical NP diagnosis was not fully proved with multiple-center studies, which will be conducted in our following study. Another issue we needed to address is that the integral role of m6A-related proteins, besides METTL3, was needed to be clarified, such as METTL14, ALKBH5, and hnRNPA2B1. We believe that these proteins could be critical for understanding the role of RNA methylation in NP progression, and their roles in NP progression could be an interesting topic in our future studies.

In conclusion, our study revealed that METTL3/YTHDF2-mediated m6A modification regulates NP progression via modulation of miR-150 processing and further suppressing BDNF expression. Moreover, serum METTL3 was downregulated in NP patients and may serve as a promising diagnostic biomarker. Our study uncovers strong evidence of METTL3-mediated m6A methylation in NP inhibition, which provides potential diagnostic and therapeutic targets for NP treatment.

## Materials and methods

### Patient samples

From October 2018 to December 2020, serum samples from 45 patients with Shingles-related NP and 49 healthy population were obtained, which were used for the detection of miR-150, YTHDF2, and METTL3 mRNA. The inclusion and exclusion criteria include: (1) 24–76 years old, (2) patients diagnosed with NP, and VAS scoring more than 4 points. In addition, patients suffering from hypothyroidism or amyloidosis were not included. Detailed information of enrolled patients was presented in Supplementary Table S1. The study was approved by the medical ethics committee of The Second Hospital, Cheeloo College of Medicine, Shandong University, and informed consent was obtained from all participants.

### Animal treatments

Animals used in this study were Sprague-Dawley rats weighing 180–200 g, obtained from Model Animal Research Center of Nanjing University. The total number of rats used in this study was 120, and estimated according to group numbers and rat number in each group. According to our established protocols [17], the SNI animal model was established to simulate the NP process. Based on our study design, the enrolled animals were blindingly grouped and received the following treatments: ①Sham-NC vectors, ②Sham-sh-METTL3, ③Sham-sh-METTL3 + miR-150, ④Sham-sh-METTL3 + Lv-YTHDF2, ⑤Sham-sh-METTL3 + sh-BDNF, ⑥Sham-anti-miR150, ⑦Sham-anti-miR150+sh-BDNF, ⑧SNI-Lv-METTL3, ⑨SNI-Lv-METTL3 + anti-miR-150, ⑩SNI-Lv-METTL3 + sh-YTHDF2, ⑪SNI-Lv-METTL3 + Lv-BDNF, ⑫SNI-miR150, ⑬SNI-miR150+Lv-BDNF. The pain behaviors were detected in the respective time points and the expressions of METTL3 and miR-150 were determined at day 14 after the rats were sacrificed. This animal experiment was authorized by the Animal Experiment Committee of The Second Hospital, Cheeloo College of Medicine, Shandong University.

### Cell culture

The rat neuron cell line RN-sc was purchased from ScienCell (ScienCell Research Laboratories Inc. catlog. #R1590, Carlsbad, CA). Cells were cultured in neurobasal medium (Thermo Fisher Scientific, Waltham, MA, USA) at 37 °C in a humidified atmosphere with 5% CO_2_. The cell characteristics were authenticated by a short tandem repeat (STR) profiling method. For in vitro cell transfection, lipofectamine 2000 was used to dysregulate the respective transcripts.

### Spared nerve-injury (SNI) surgery

The male rats were anesthetized by intraperitoneal injection. Rats were fixed in the prone position on the rat holder and exposed the left lower limb of the rat, followed by sterilizing with iodine and alcohol three times. Then, a longitudinal incision ~6–8 mm long was made behind the left thigh to expose the subcutaneous muscles. After bluntly separating the biceps femoris, semitendinosus muscle, and semimembranosus muscle with a hemostatic forceps, the sciatic nerve was isolated, and the left foot twitched after stimulation. Afterward, the sciatic nerve trunk was clamped with a large hemostatic forceps 6–8 mm away from the ischial tuberosity, squeezed for 5 seconds and then released, and repeated 3 times. After marking with 9-0 noninvasive sutures, the sciatic nerve was returned to the original position, organized the muscles, and sutured the wound. For sham group, the rats received similar treatments and only the skin is cut to uncover the left sciatic nerve without clamps.

### Intrathecal catheter implantation and lentivirus injection

METTL3, miR-150, and BDNF were stably dysregulated via constructing lentivirus-based vectors by GenePharma (Shanghai, China) and injected with an intrathecal catheter-implantation method [18]. The treatment timeline was shown in Supplementary Fig. S1, and sequences for silencing vectors were presented in Supplementary Table S2.

### NP behavior assessment

The value of paw-withdrawal threshold (PWT) was evaluated to show the degree of mechanical allodynia. A transparent plastic box implanted with metal receptors was applied and the von Frey filament (North Coast Medical, Gilroy, CA, USA) was used to touch the toe skin of the hind limbs. Data were recorded when the paw was identified. The number of spontaneous flinches (NSF) was recorded to evaluate the spontaneous pain severity according to the number of spontaneous hind limb flinching of the right hind paw.

### RNA extraction and quantitative PCR

According to the instructions of the manufactures, the total RNAs from spinal cord tissues or RN-sc cells were separated by using TRIzol reagent (Invitrogen, Carlsbad, CA, USA). RNA was transcribed into cDNA using the RevertAid First Strand cDNA short Kit (Thermo Fisher Scientific). The reaction conditions for reverse transcription were 38°C for 40 min followed by 82°C for 10 s. PCR amplification was performed with a SYBR Green method with cDNA as a template using the respective primers (Biosune Biotech, Shanghai). GAPDH was used as an endogenous control for METTL3 and miR-150. The relative expression is calculated through 2-^ΔΔCt^ method. The detailed sequences of the respective primers were listed in Supplementary Table S2.

### RNA immunoprecipitation (RIP)

RIP analysis was performed with the Magna RIP RNA-binding protein immunoprecipitation kit as per the manufacturer’s protocol (Millipore, Bedford, MA). Briefly, samples were irradiated at 254 nm, 400 mJ/cm^2^ (Stratagene Stratalinker), followed by treatment with RIP lysis buffer. The immunoprecipitation was implemented using the with antibodies against m6A (1:800, ab208577, Abcam, Cambridge, MA), DGCR8 (1:800, ab90579, Abcam, USA), and YTHDF2 (1:1000, cat. no. ab246514, Abcam, USA) proteins. The enriched RNA was analyzed via qRT-PCR.

### Co-immunoprecipitation (Co-IP) assay

To verify the direct binding between METTL3 and DGCR8, RN-sc cells were lysed and incubated with antibodies against METTL3 (ab195352, Abcam, USA) at 4 °C overnight. Subsequently, cell lysates were cultivated with protein A/G agarose beads (Santa Cruz, CA, USA) for 2 h. Finally, western blotting with antibodies against DGCR8 (ab90579, Abcam) were employed to analyze the respective protein expression in immuno-complexes.

### Immunofluorescence staining analysis

To define the relative expression of METTL3 and its colocalization with NeuN and BDNF, we performed immunofluorescence staining using spinal cord tissues. Frozen slides bearing tissues from rats of the respective groups were incubated with primary antibodies, including anti-METTL3 antibody (1:200, ab195352, Abcam), anti-NeuN antibody (1:200, ab104224, Abcam), anti-GFAP antibody (1:200, 3670 S, Cell Signal Technology, USA), anti-IBA1 antibody (1:200, ab5076, Abcam), and anti-BDNF antibody (1:200, ab108319, Abcam) under refrigerated environment for more than 12 h. Afterward, slides were rinsed thoroughly followed by culture with the respective fluorescently labeled secondary antibodies for 2 h at room temperature. The stained tissues were captured with a Zeiss ZFM-700 fluorescence microscopy (Heidenheim, Germany).

### Reporter construction

To analyze the regulation of METTL3-mediated m6A methylation on primiR-150 processing, we constructed ectopic primiRNA reports as *Auyeung V.C*. reported [42]. Briefly, the miRNA control primiR-1-1 was modified by mutating the adenosines (A’s) of the predicted sites. The wild type pri-miR-150 or a mutant one was generated targeting thymidines (Ts). Lipofectamine 3000 (Invitrogen) was used for transfecting the above-established vectors. Forty-eight hours later, RT-qPCR was performed to quantify the expression levels of miR-150 and mature miR-1-1.

### Western blot analysis

The collection and quantification of total proteins from sample cells/tissues were carried out by RIPA lysis buffer (Beyotime, Shanghai, China) and BCA kits, respectively. After exposure to 10% SDS-PAGE, the separated proteins were loaded onto PVDF membranes, followed by blockage with 5% bovine serum albumin (BSA, Solarbio, Beijing, China). Then, the membranes were incubated with respective primary antibodies at 4 °C for more than 12 h. The antibodies used in this study include as follows: BDNF (1:1000, ab108319, Abcam), METTL3 (1:1000, ab195352, Abcam), and GAPDH (1:1000, ab8245, Abcam,), DGCR8 (1:1000, ab191875, Abcam), YTHDF2 (1:1000, ab246514, Abcam). On the next day, the membranes were incubated with secondary antibodies with the same origin. After being thoroughly rinsed with PBS buffer, the protein bands were visualized with the application of enhanced chemiluminescence (ECL, Millipore, USA). The original western blots were provided in Supplementary Material.

### RNA m6A dot-blot assays

First, the mRNAs were denatured under a high temperature of 95 °C for 10 mins followed by transferring to a Hybond-N membrane. After that, the membranes were cultured with m6A antibody (1:1000, ab208577, Abcam) at refrigerated environment for more than 12 h. Following that, a HRP-linked second antibody anti-mouse IgG (1:5000, 7076 S, Cell Signal Technology, USA) was used at room temperature for one hr. Finally, the membrane can be visualized using ECL reagent. To obtain a consistent result between groups, the membranes were stained with methylene blue. The original dot blots were provided in Supplementary Material.

### Statistical analysis

Data were analyzed with GraphPad Prism 8 (GraphPad Software, USA). The chi-square test was employed to verify the association between METTL3 expression levels and other molecules clinicopathological characteristics. The Pearson linear-regression analysis was used to determine the expression relationships. The statistical analysis between the two-group data was compared by t-test. One-way analysis of variance was employed to compare the differences between multiple groups of data, and then the Tukey post hoc test was utilized to analyze between the two groups of data. *P* < 0.05 indicated statistical significance. Data were presented as mean ± SD values. All experimental assays were conducted in replicates. Data conformed to normal distribution and variance was mild within each group of data.

## Supplementary information

Supplementary Material

## Data Availability

The datasets used and/or analyzed during the current study are available from the corresponding author on reasonable request. The supplementary material is available at the official website of the journal.
