# Supplementary material for: METTL3 suppresses neuropathic pain via modulating N6-methyladenosine-dependent primary miR-150 processing
Source: Cell Death Discov. 2022 Feb 24;8:80. doi: 10.1038/s41420-022-00880-2 (PMC8873433; doi:10.1038/s41420-022-00880-2)

Supplementary Materials

Supplementary figures

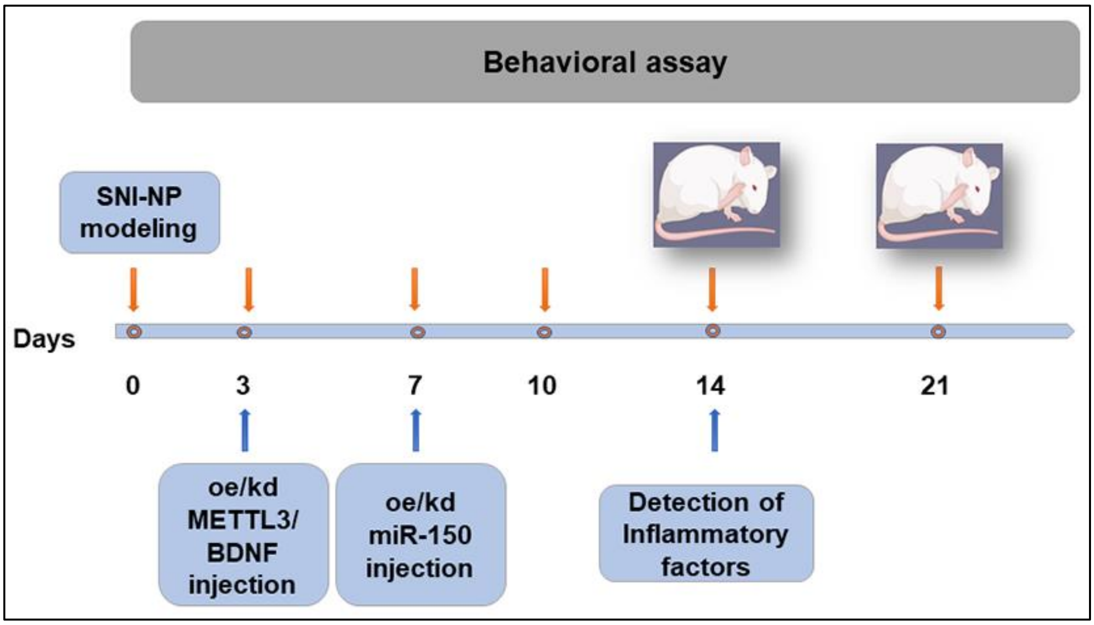

Supplementary Figure S1. The flow chart of manipulations of respective lentivirus vectors in SNI rats.

```
Rattus norvegicus strain BN/NHsdMcwi chromosome 1, mRatBN7.2, whole
genome shotgun sequence

CTTGTGTTCCTCAGGTGTGCAAAGAAGGCCCAAGACCACAATCTCTACTCCGCAGATGATACAACTGCC
TAAGGGTCGTCAGGGGTCTTTCTTTTCTCAGGTGTCCCCTCACAGGCTCTGAAAATTTGAAAGGCGAAG
CCCCAACCACCCCTGGGAGGAAACAAGGGAGCTGGGCTGTTGTTGTAAACAGAGCCCCACCCGGAACGG
AAGCACCTGCGGCTGGAGGGGGCCACACCTCTGAACAATTGGCTTCAGATTCCTCAGGGTCCCTGTCCC
CCGTAGCTCTGAGGCTAGTCCCTCCTCTGCTGGGTGTCTCCACTCCTGGGTGGCTGGCAGGCGCCCAGC
TTGGGCGGTGCCCCCTCCTGACCGGTCCCTGCCCCTTCCTGCTCGCTTTGATGCAGGCCCACTTCCTTT
GACAGGAACCCCCTCCCTCAGCGGCCTGGTAAAAGGCAGAAGGCCGTGGTGAAGCGGTGCATTCCGCAG
CATCTCTGCAGAGGACT     premiR-150
CTTCTCAAGGCCCTGTCTCCCAACCCTTGTACCAGTGCTGTGCCTCAGACCCTGGTACAGGCCTGGGGG
ACAGGGACTTGGGGAC
```

Supplementary Figure S2. M6A motifs in the primiR-150.

Supplementary Tables

Supplementary Table S1 Characteristics of patients with NP and the healthy control group (CG)

Characteristic	NP (N=45)	CG (N=49)	Statistical Significance
Age (y; mean±SD)	57.4±12.4	56±12.8	P > 0.05
Gender (n; female/male)	26/19	29/20	P > 0.05
BMI (kg/m^2; mean±SD)	23.1±3.1	22.6±3.6	P > 0.05
HZ Location (n/N)			
• Thorax	21/45	-	-
• Abdomen	9/45	-	-
• Extremities	7/45	-	-
• Other	8/45	-	-
Pain VAS (0-10; mean±SD)	6.0±1.7	-	-

Supplementary Table S2. Information of the sequences of qPCR primers and silencing oligonucleotides

qPCR primer name	Sequence (5’-3’)
METTL3 (Forward)	CACACTCCTCGGGGTTTGAT
METTL3 (Reverse)	ACAGTTAGTGGCGTGAGAGC
BDNF (Forward)	GCCAGGGGCAACTCATCTTC
BDNF (Reverse)	GGTTGAAAGGCGCAGATGTC
MiR-150 (Forward)	CTTCTCAAGGCCCTGTCTCC
MiR-150 (Reverse)	TCCCCAAGTCCCTGTCCC
YTHDF2 (Forward)	CCTTAGGTGGAGCCATGATTG
YTHDF2 (Reverse)	TCTGTGCTACCCAACTTCAGT
GAPDH (Forward)	AGTTAATGCCGCCCCTTACC
GAPDH (Reverse)	CAGGGCTGACTACAAACCCA
U6 (Forward)	CTCGCTTCGGCAGCACA
U6 (Reverse)	AACGCTTCACGAATTTGCGT
**RIP qPCR primer**	**Sequence (5’-3’)**
primiR-150 Forward	GTGGTGAAGCGGTGCATTCC
primiR-150 Reverse	CACTGGTACAAGGGTTGGGAG
**shRNA name**	**Sequence (5’-3’)**
sh-METTL3 shRNA	CCUGCAAGUAUGUUCACUA
sh-YTHDF2 shRNA	CCTACTTACCCAGTTACTACA
sh-BDNF shRNA	CCGGCCGGCATTGGAACTCCCAGTGTTCAA-<br>GACGCACTGGGAGTTCCAATGCCTTTTTG
Scrambled control	CAGGTGGACTCACAATTCCAAATAT
miR-150 inhibitor	GACCAUGUCCGGACCCCCU
Inhibitor NC	CAGUACUUUUGUGUAGUACAA

Original western blots

Fig 1j

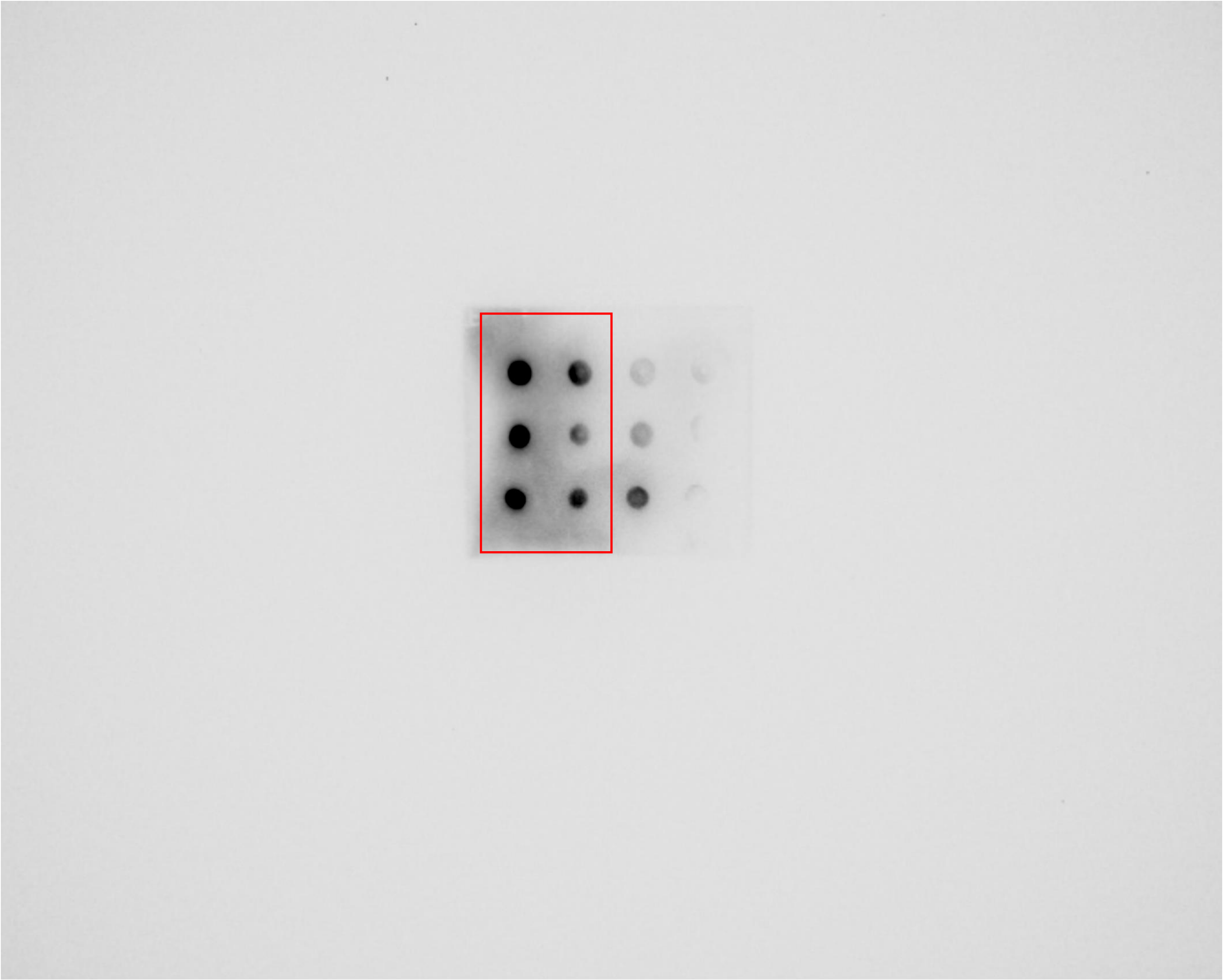

Fig 1j

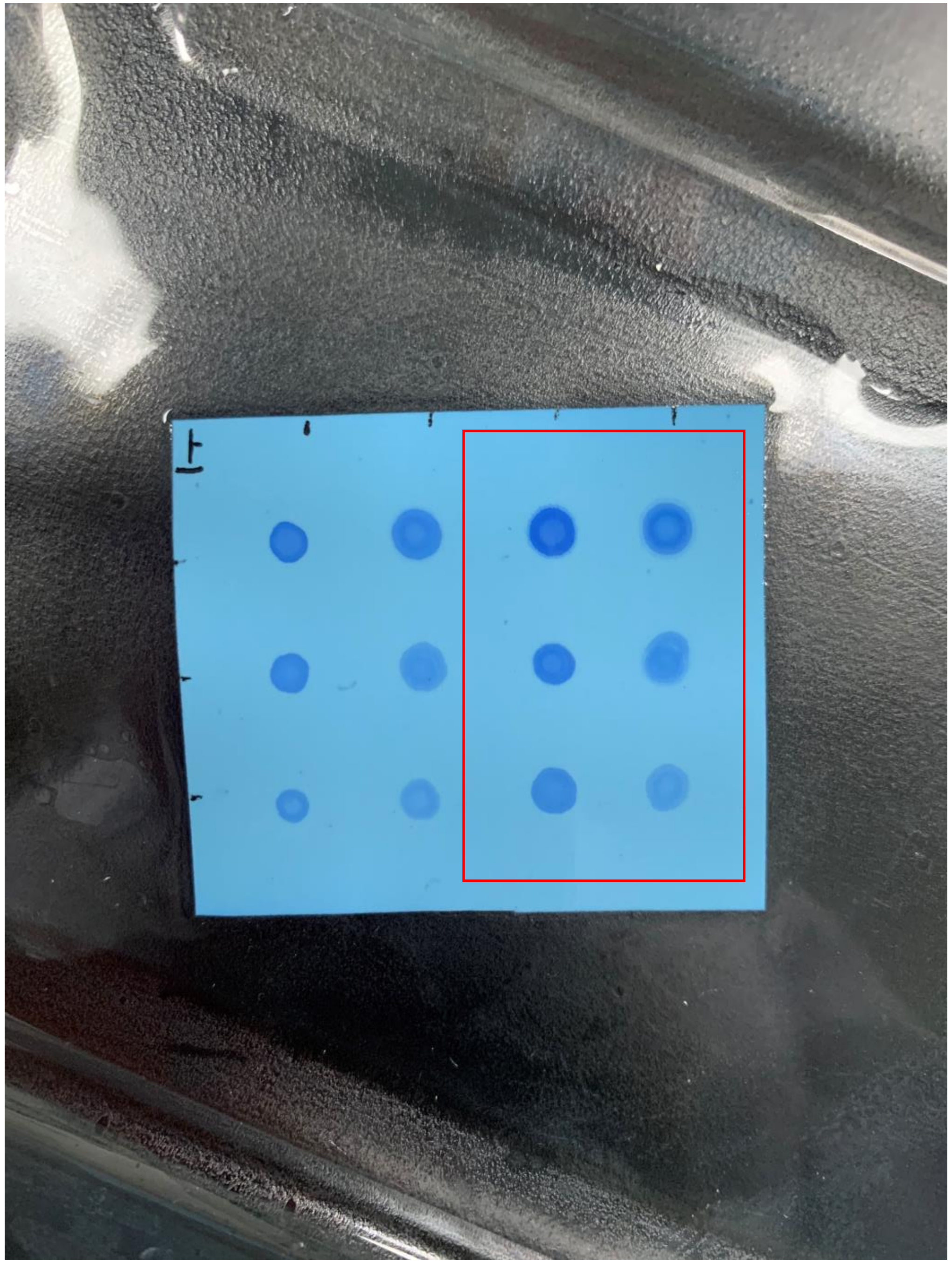

Fig 2b

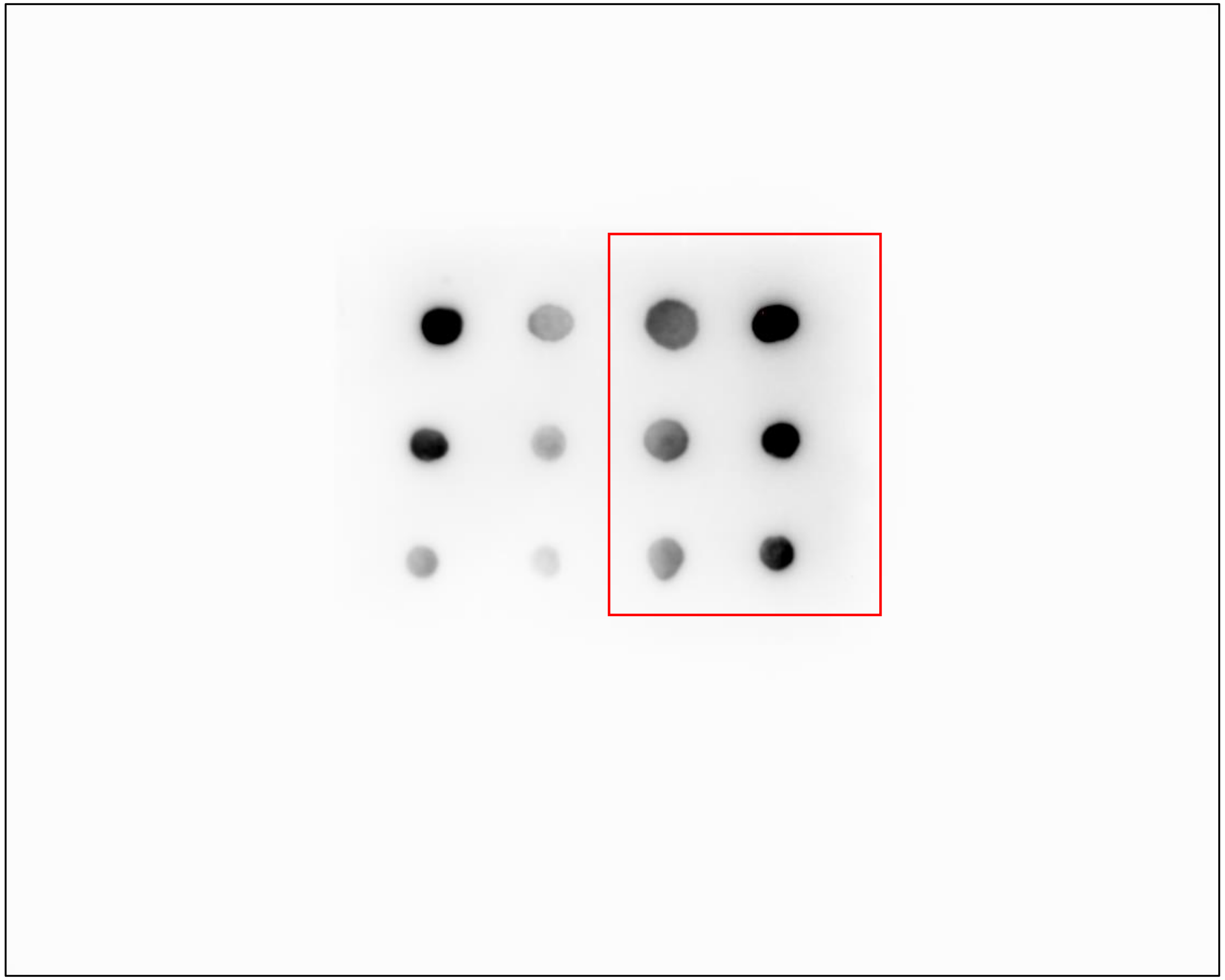

Fig 2b

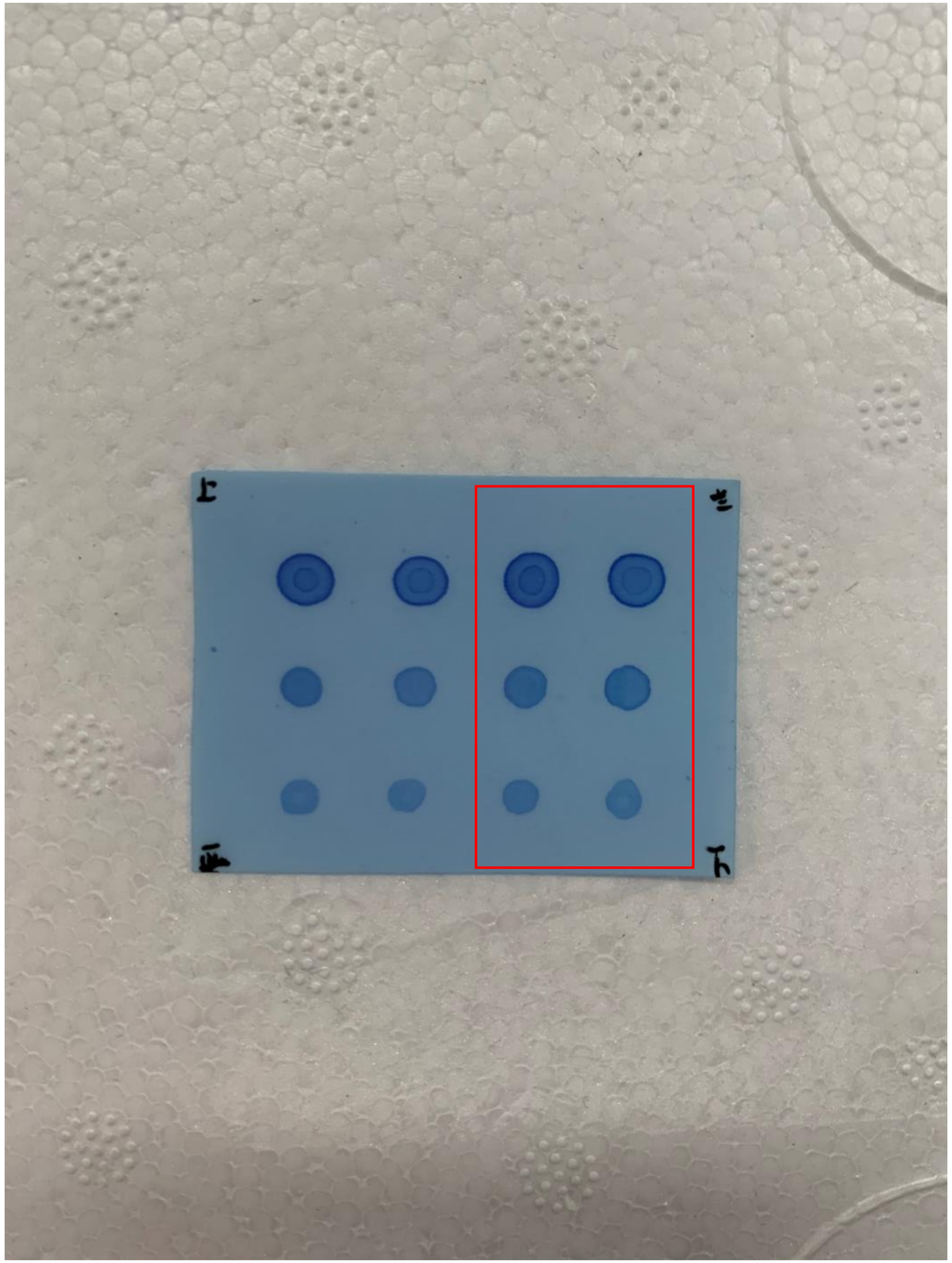

Fig 2g

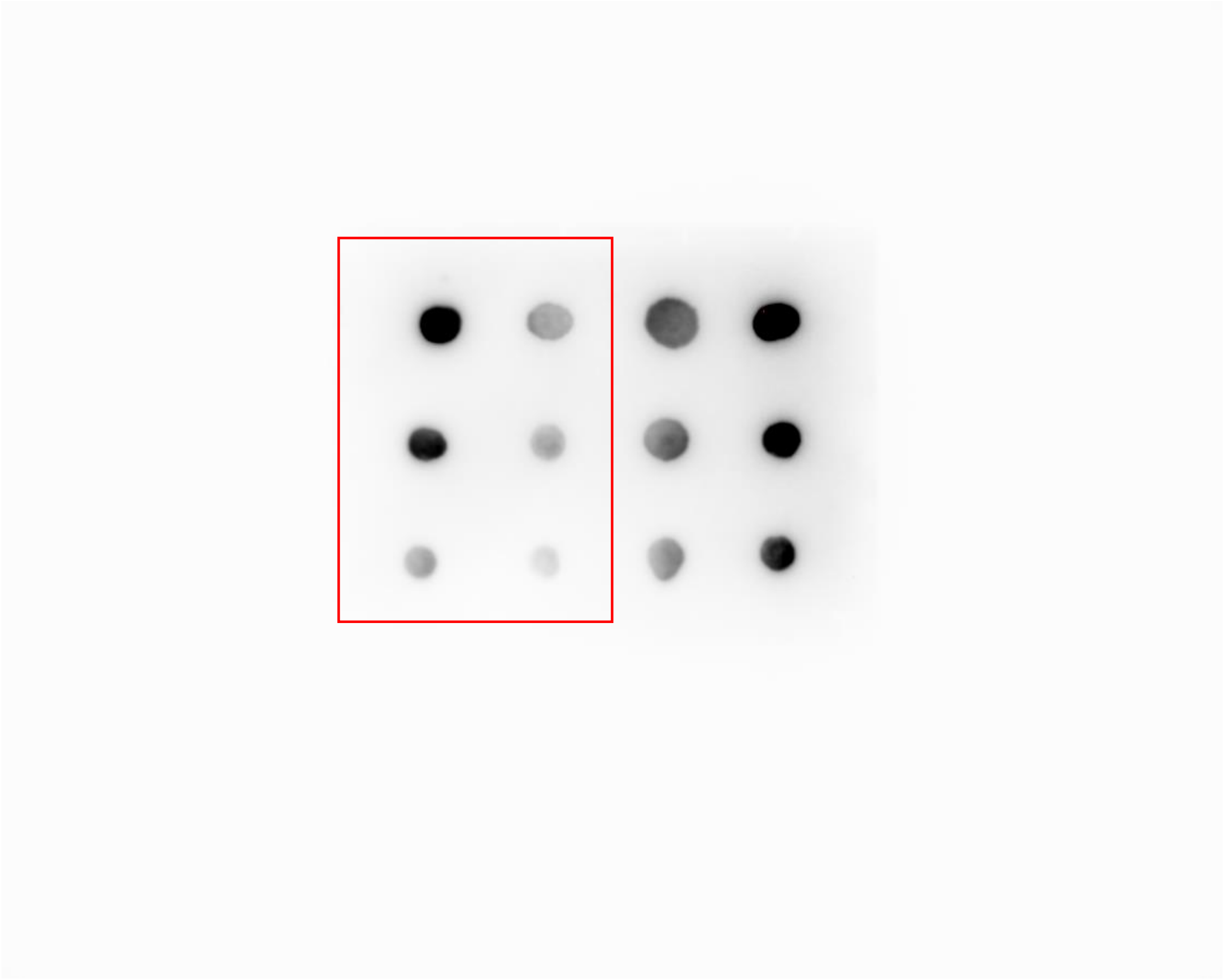

Fig 2g

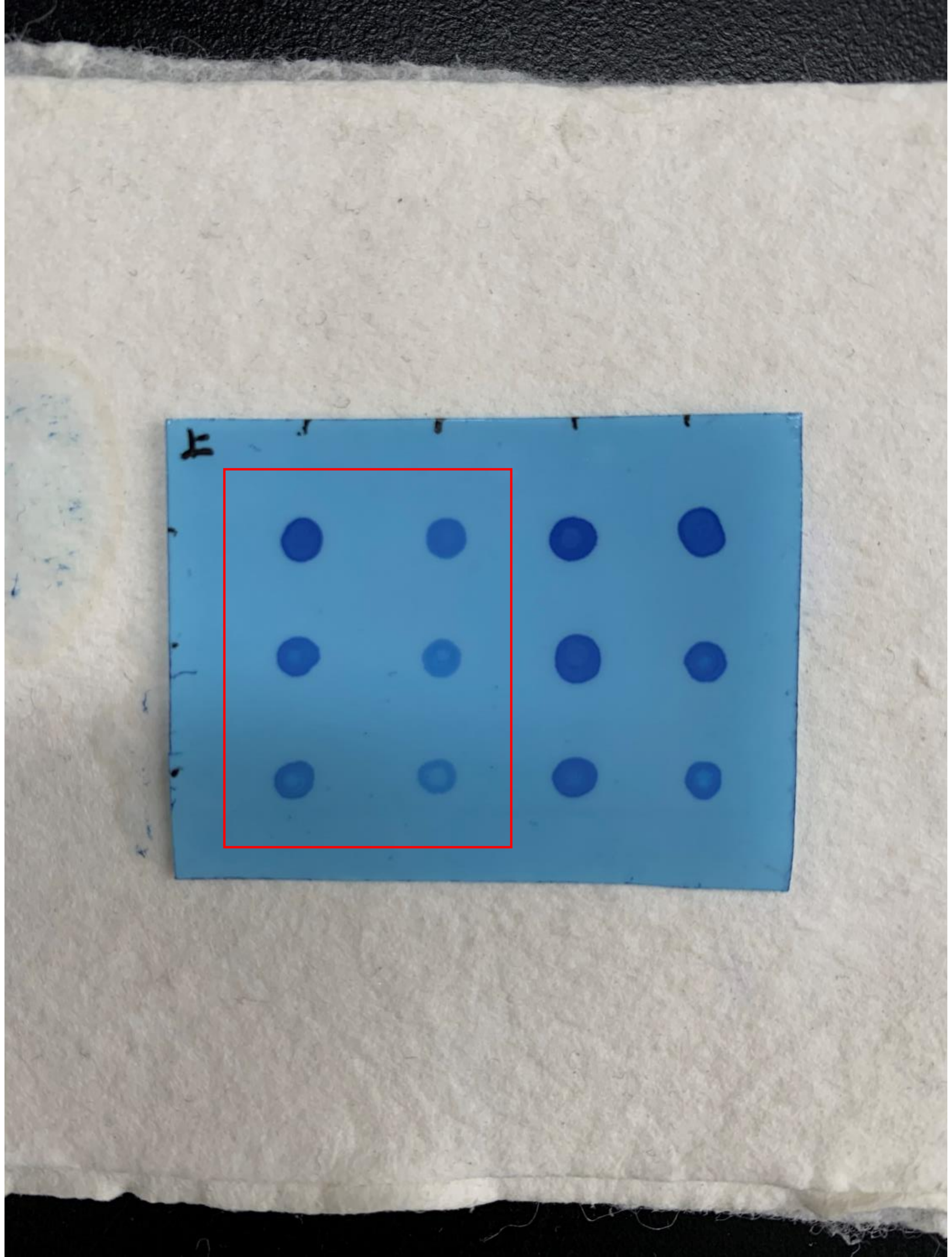

Fig 3a

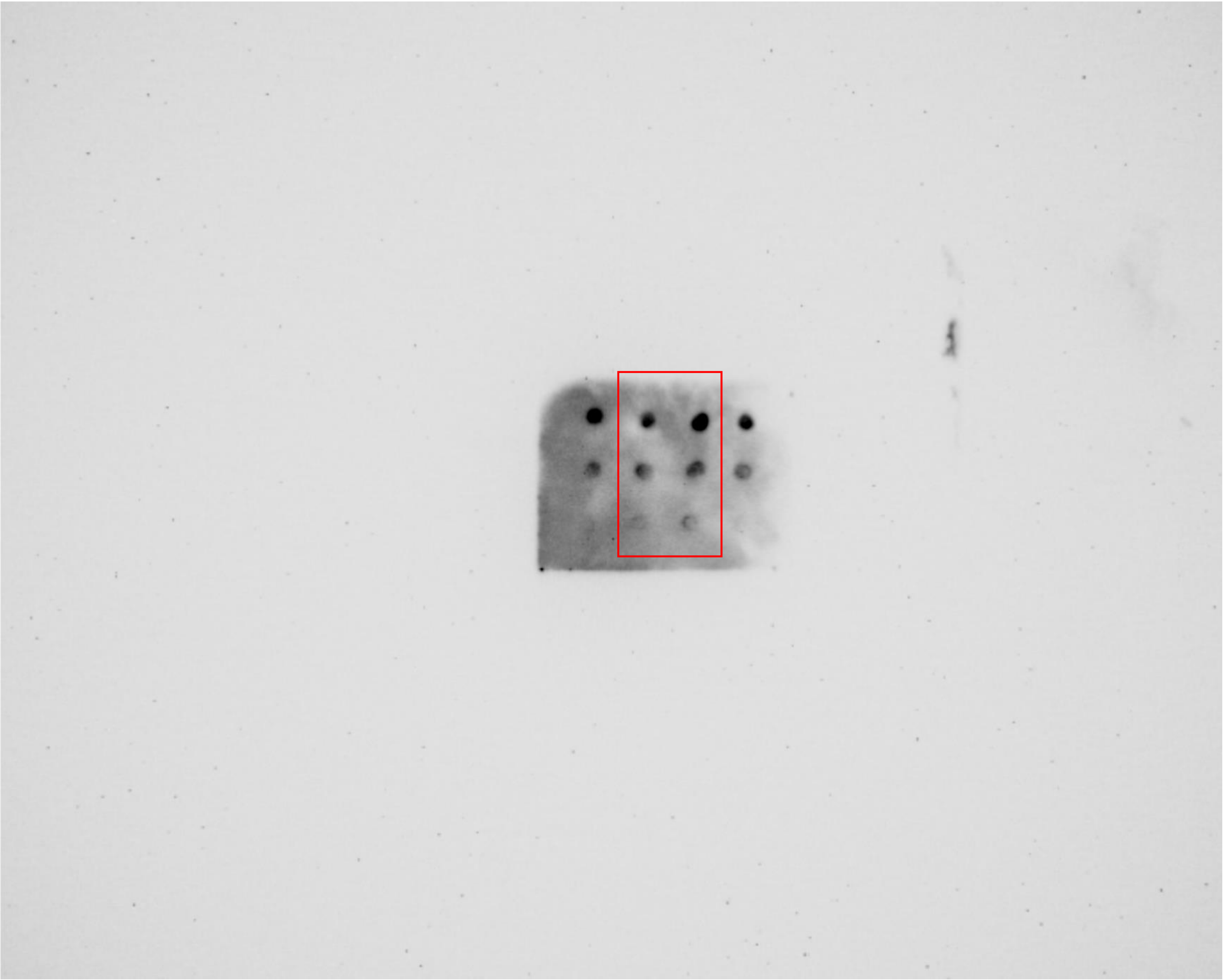

Fig 3a

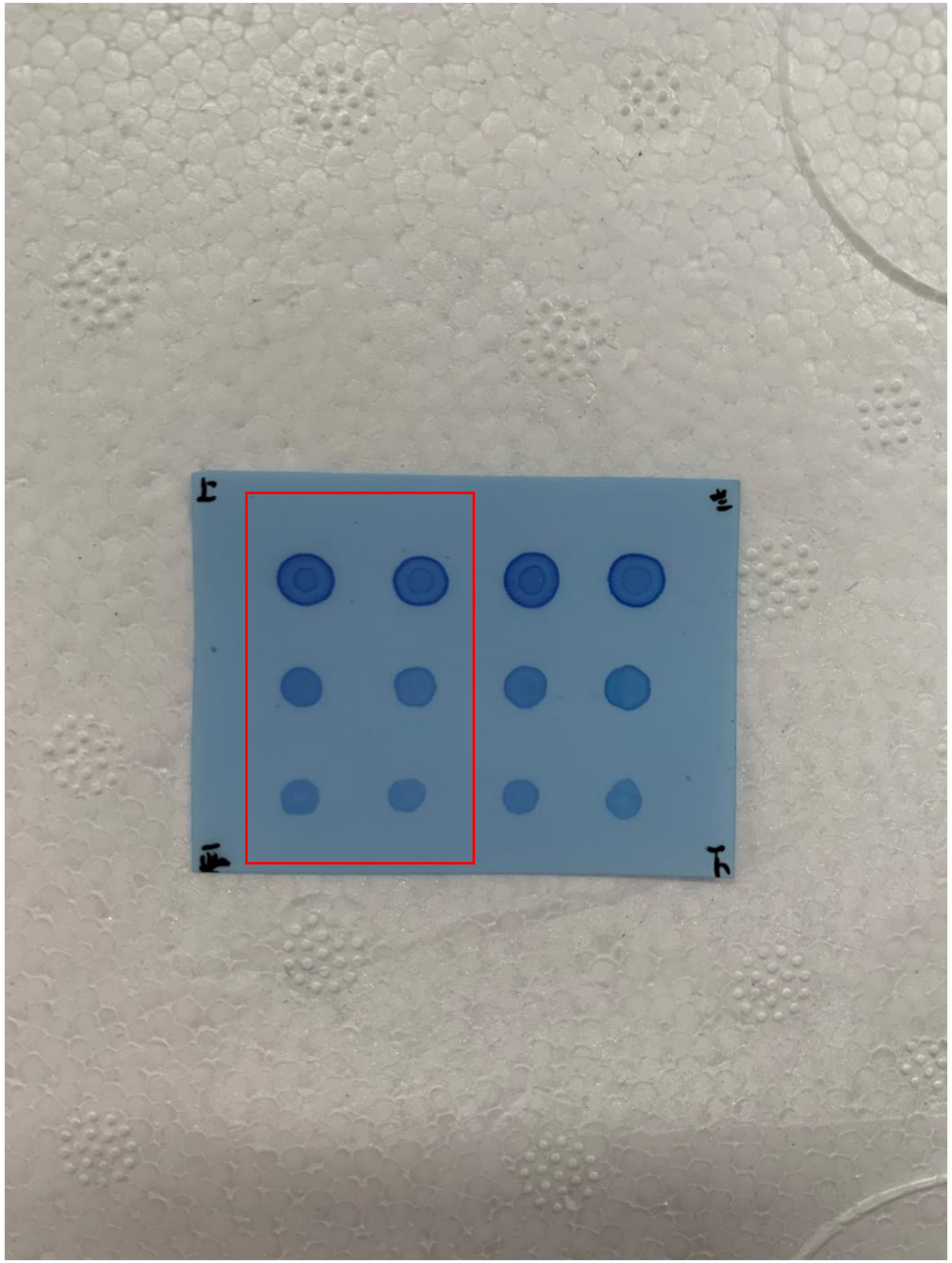

Fig 3b

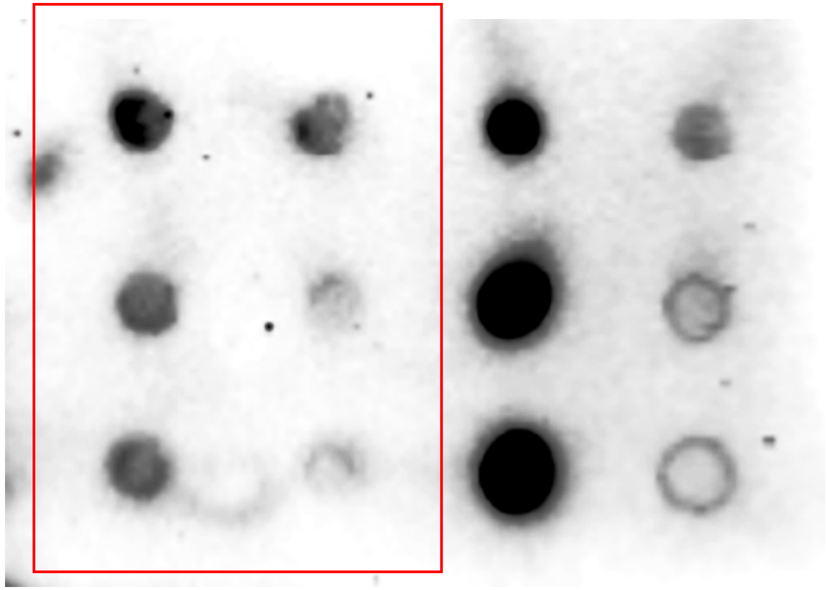

Fig 3b

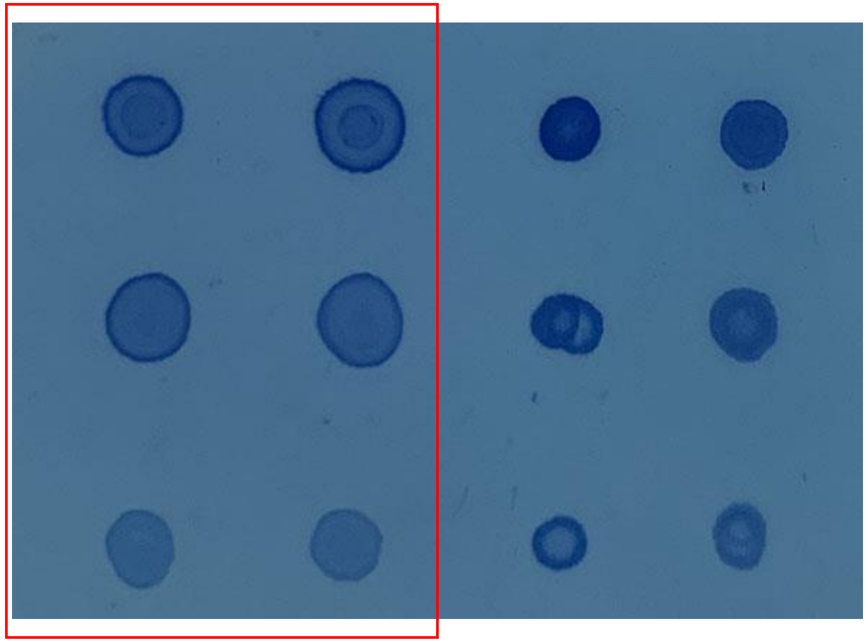

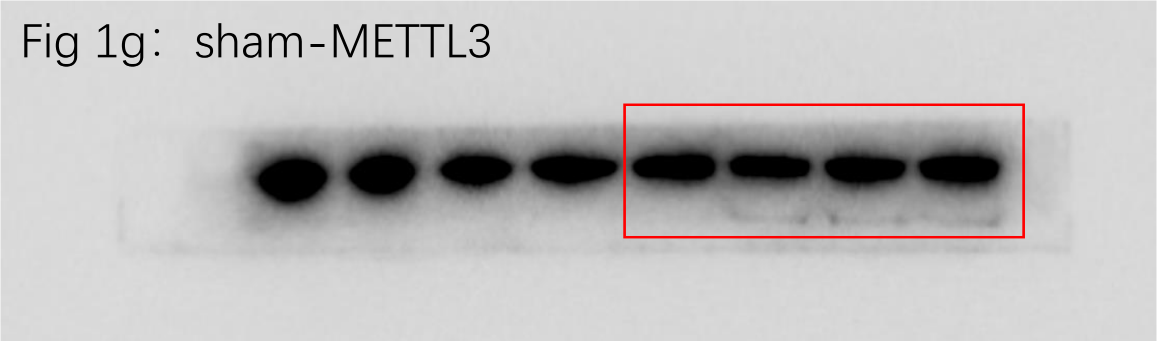
Fig 1g：sham-METTL3

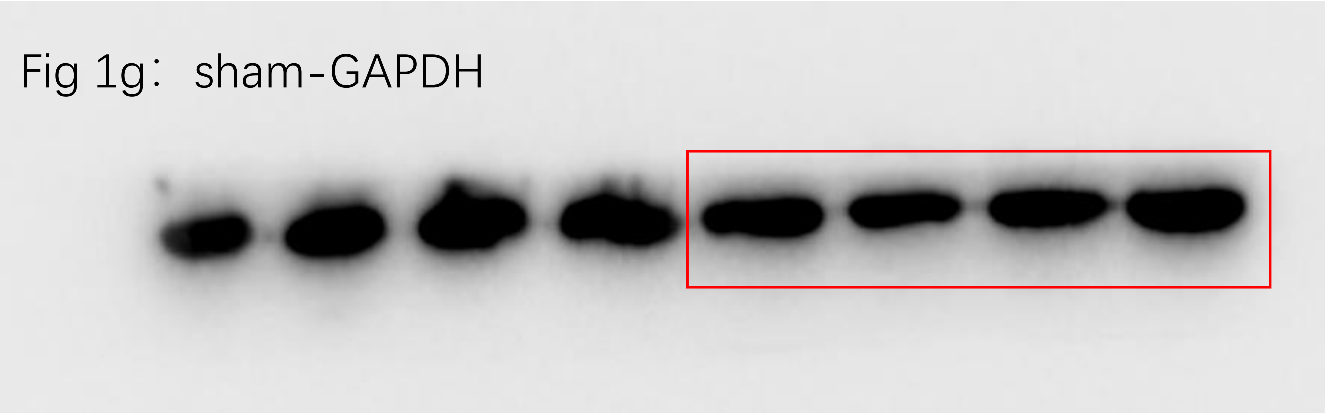
Fig 1g：sham-GAPDH

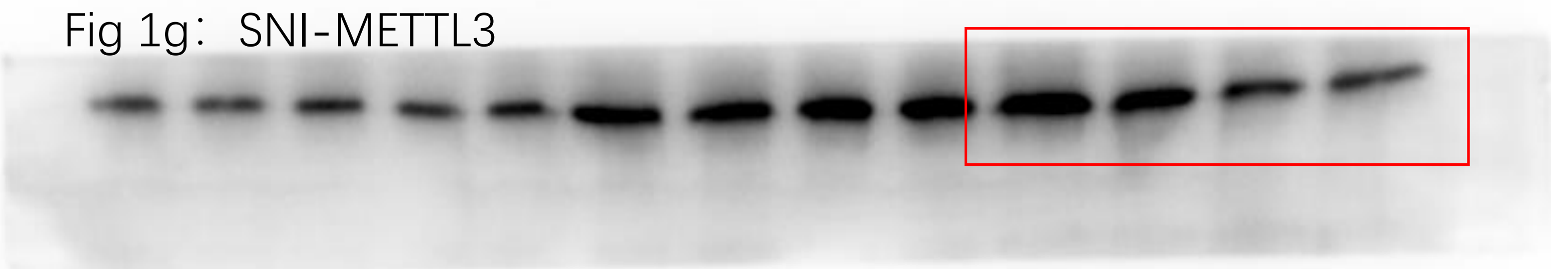
Fig 1g：SNI-METTL3

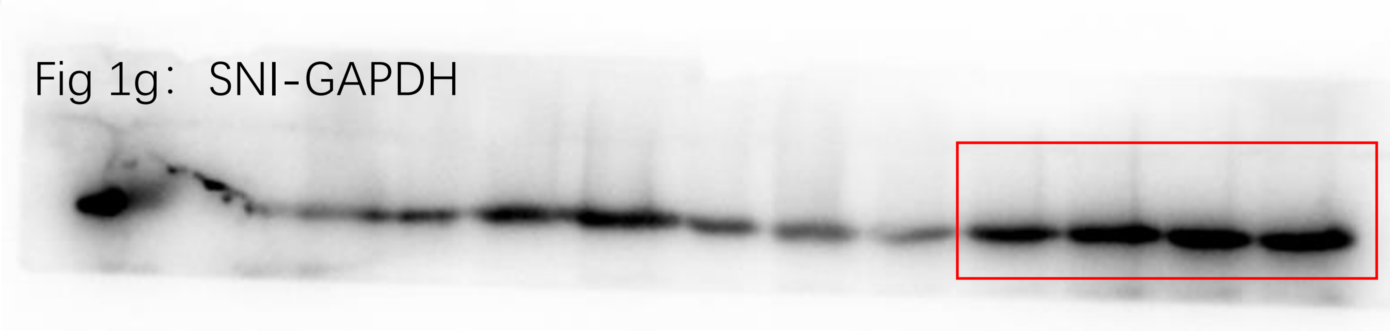
Fig 1g：SNI-GAPDH

Fig 2a： mettl3

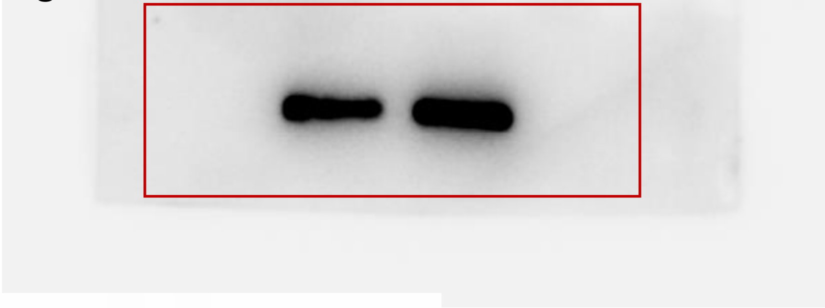

Fig 2f： gapdh

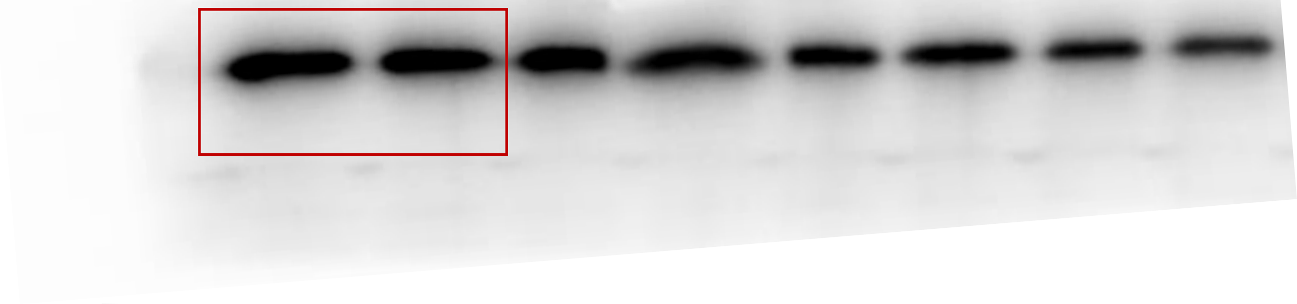

Fig 2a： gapdh

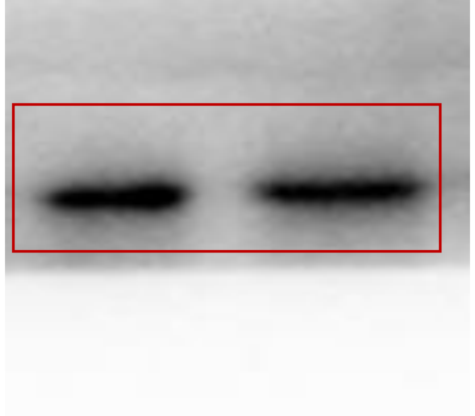

Fig 2f： mettl3

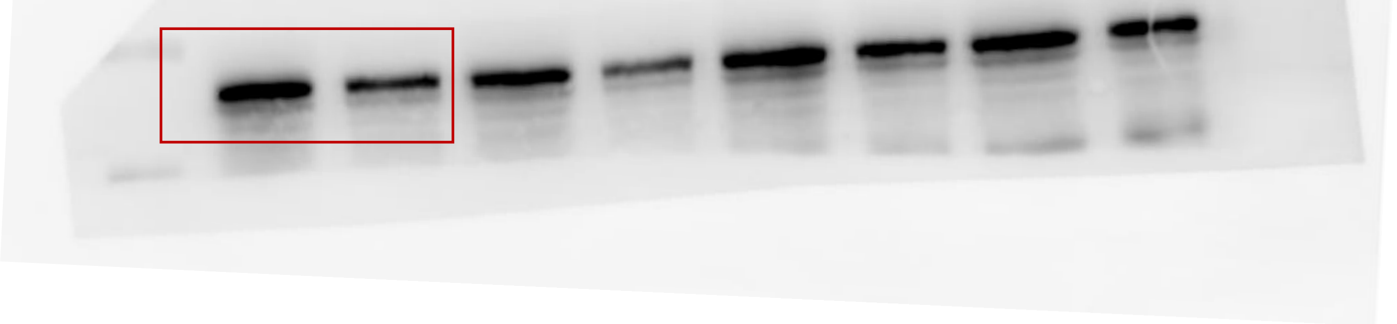

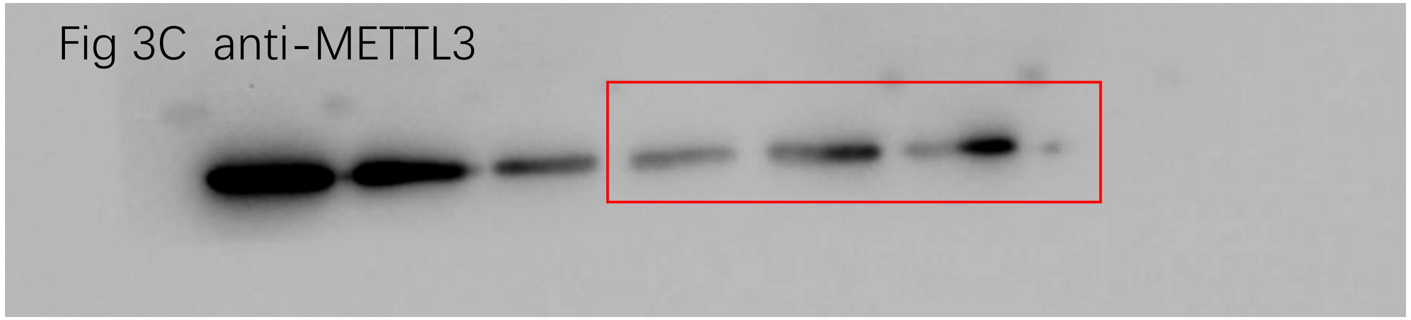
Fig 3C anti-METTL3

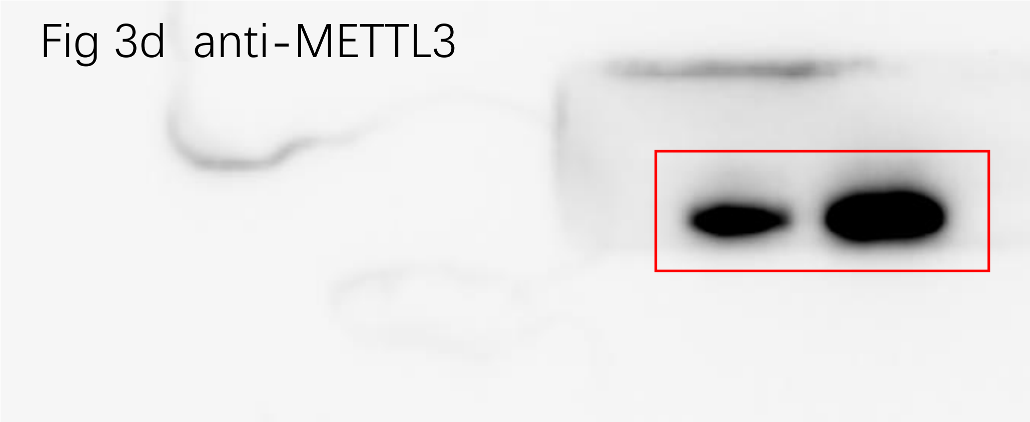
Fig 3d anti-METTL3

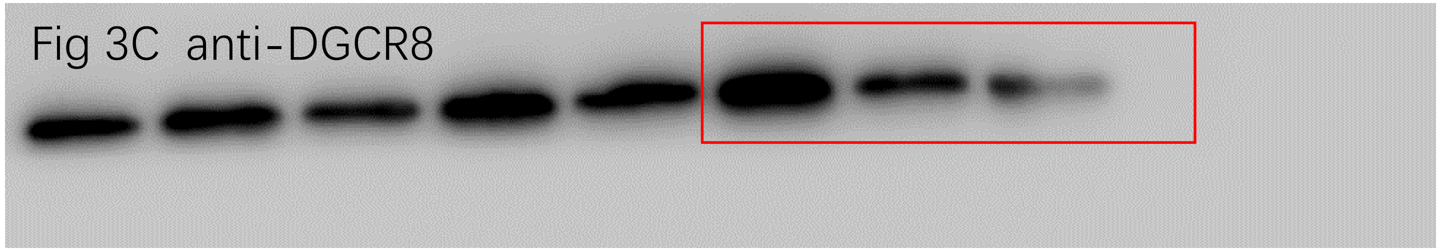
Fig 3C anti-DGCR8

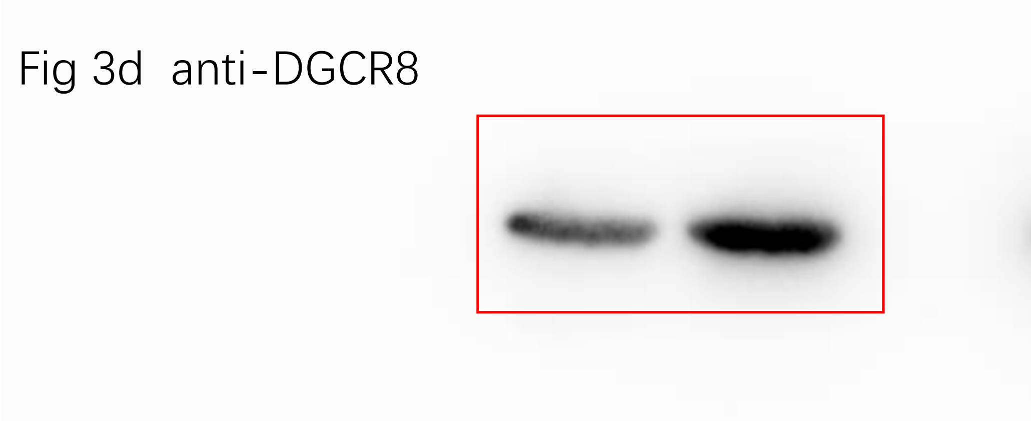
Fig 3d anti-DGCR8

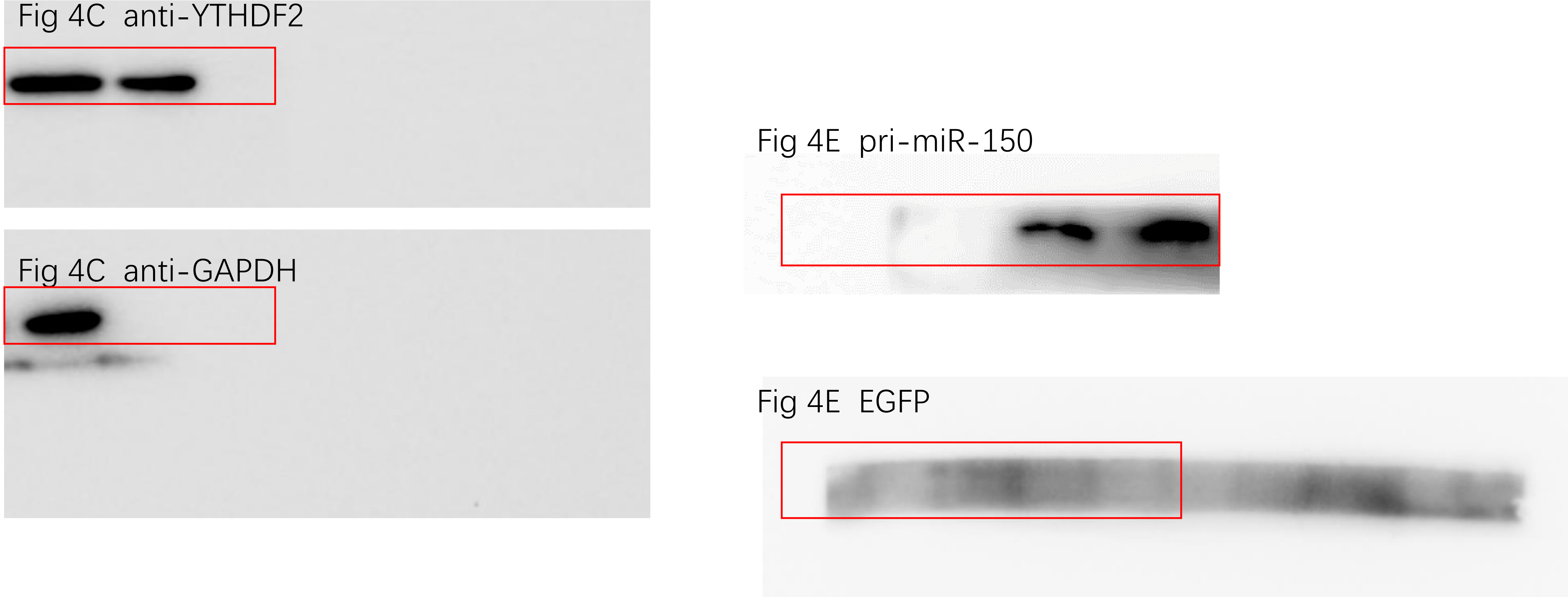
Fig 4C anti-YTHDF2
Fig 4C anti-GAPDH
Fig 4E pri-miR-150
Fig 4E EGFP

Fig 4G

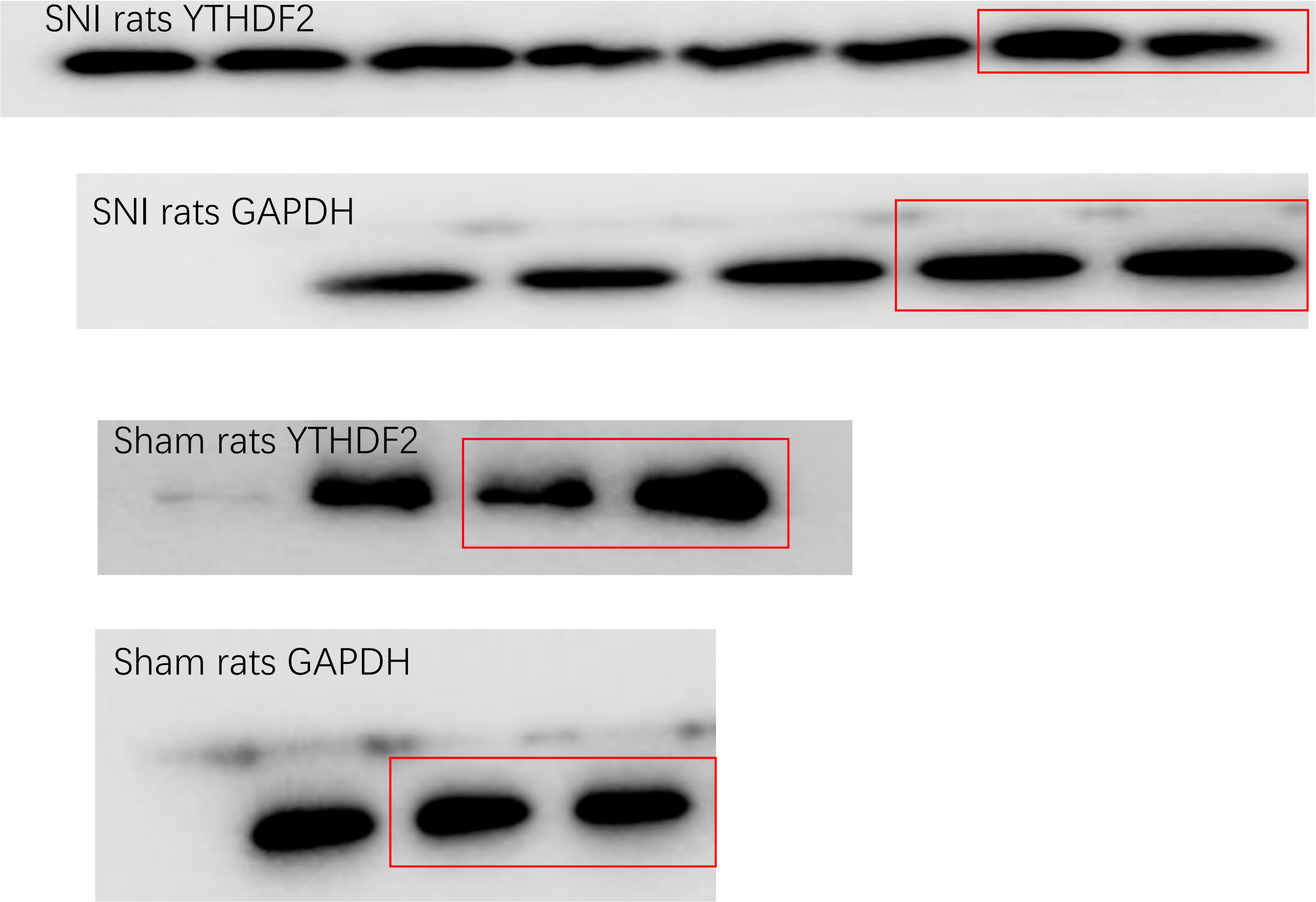
SNI rats YTHDF2
SNI rats GAPDH
Sham rats YTHDF2
Sham rats GAPDH

Fig 6C

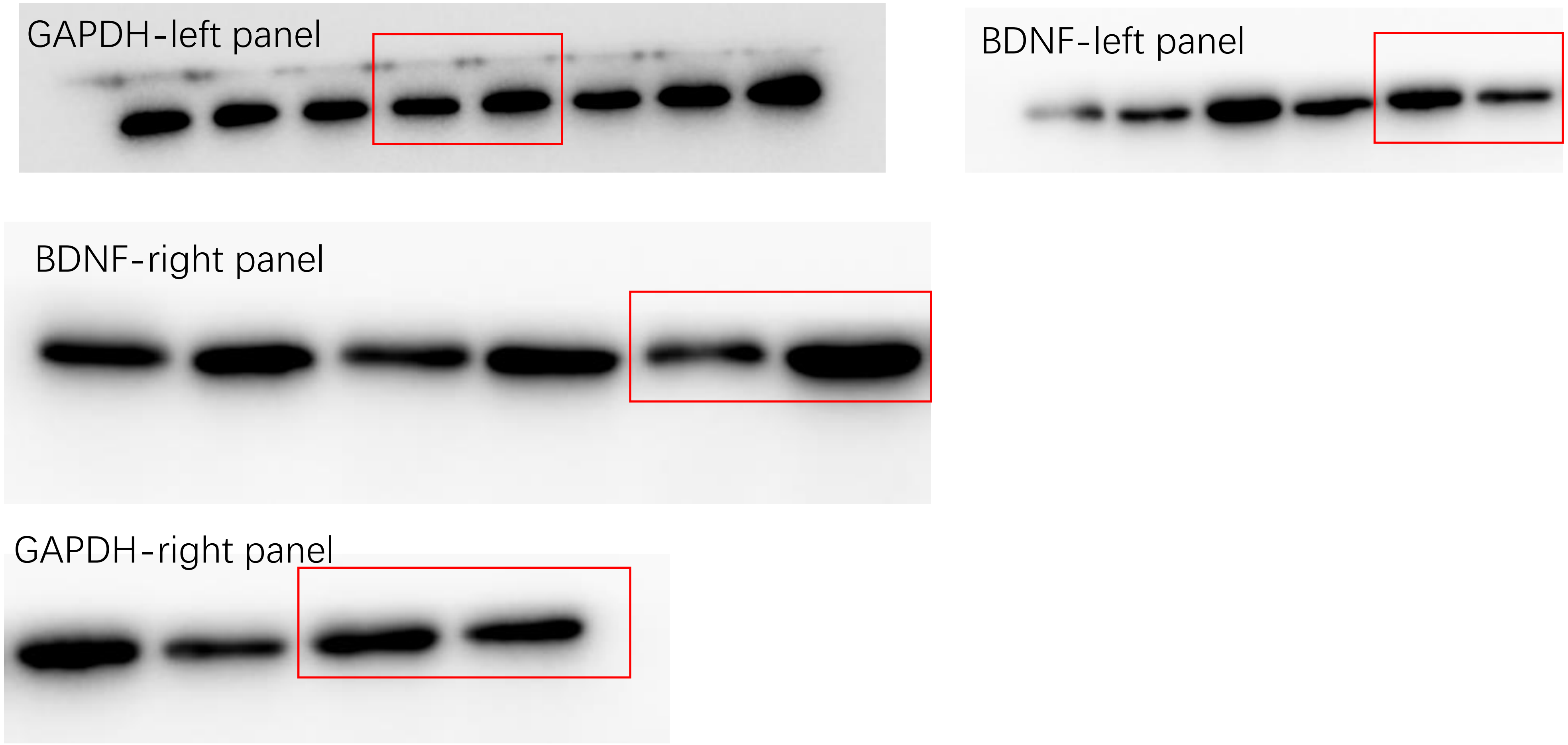
GAPDH-left panel
BDNF-left panel
BDNF-right panel
GAPDH-right panel

Fig 6d

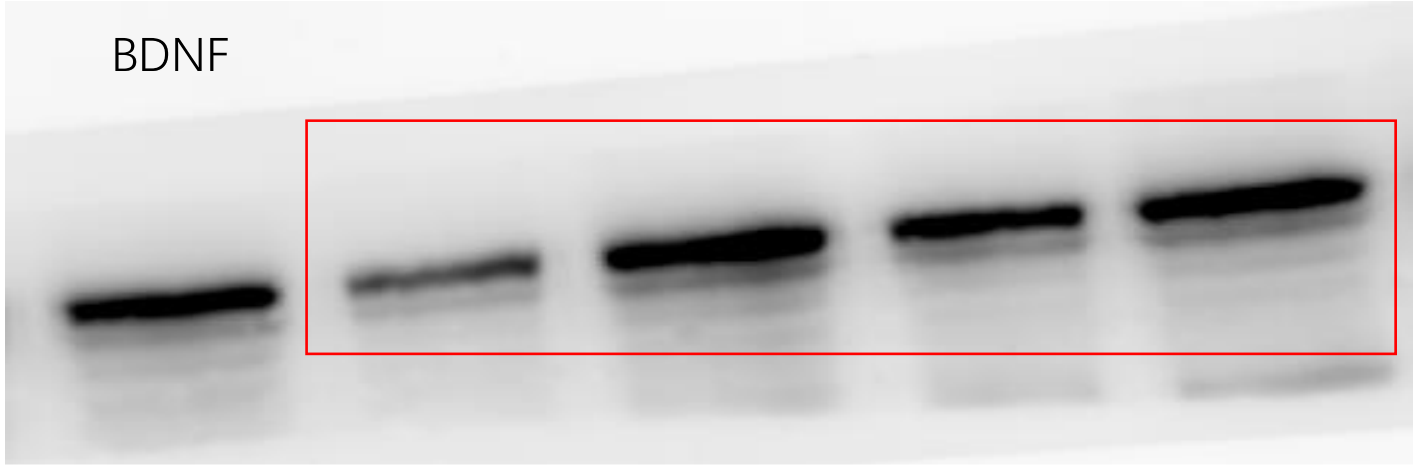

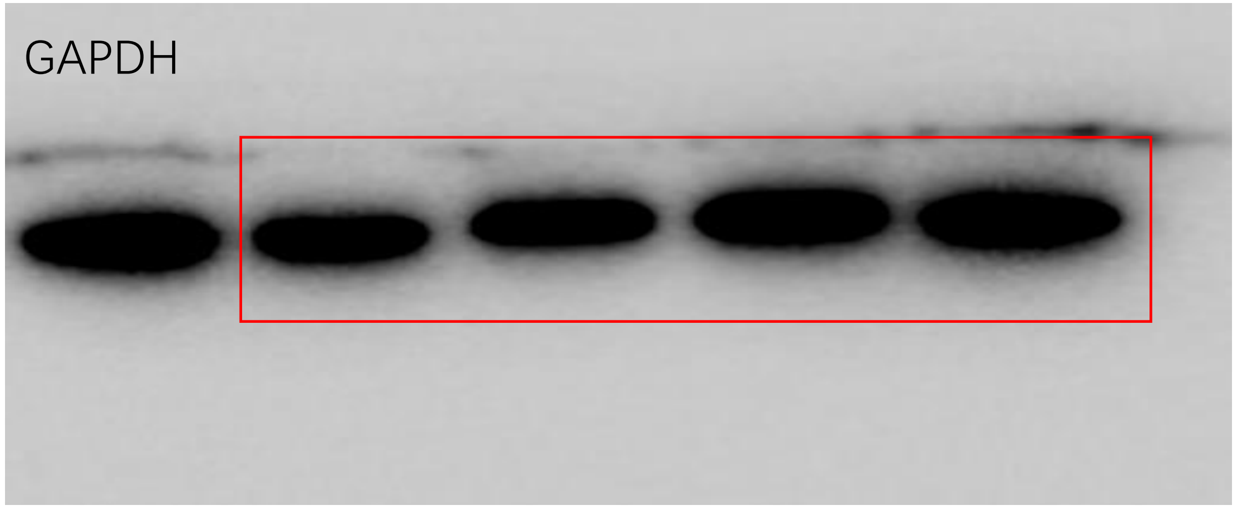

Fig 6g

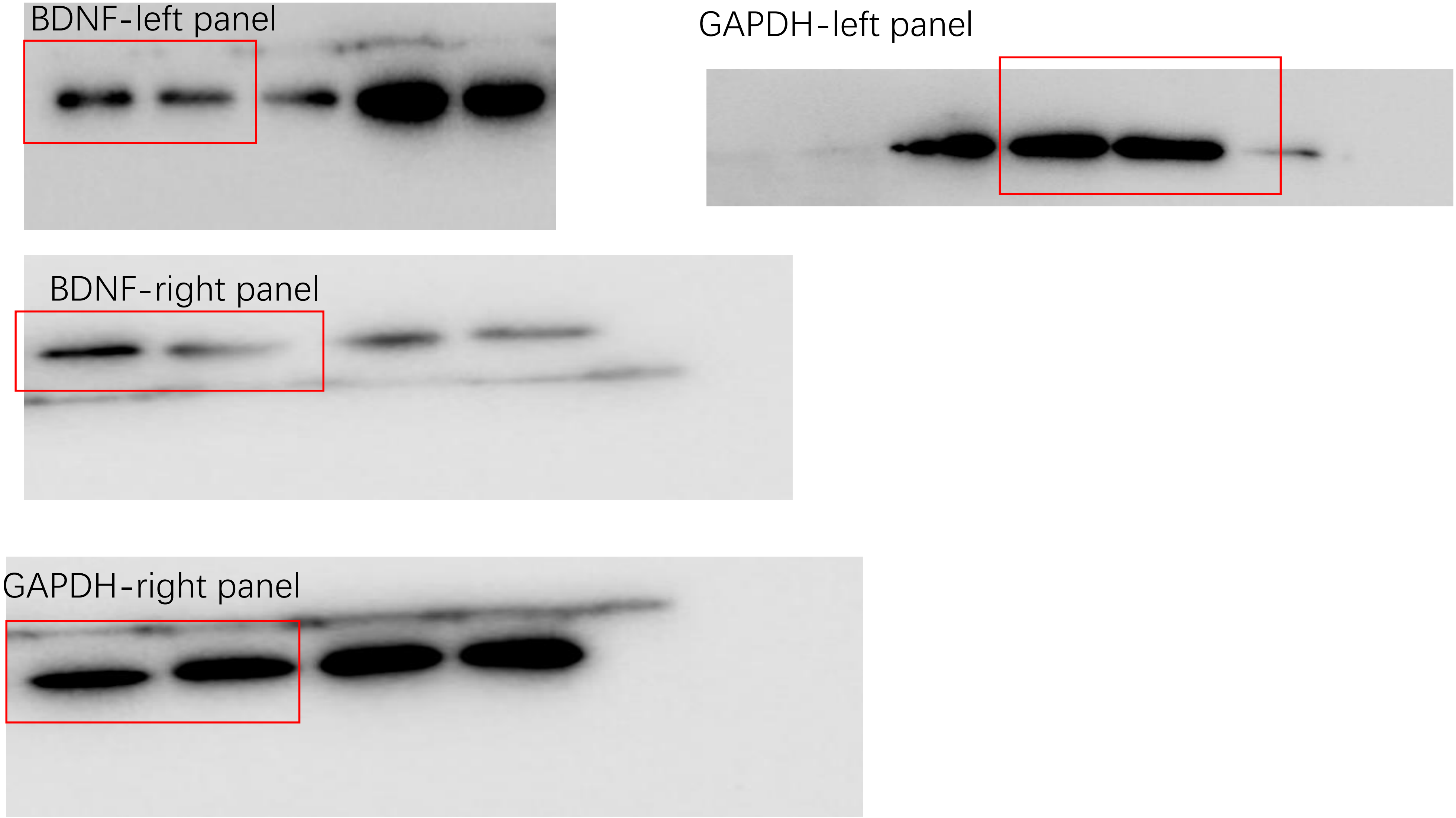
BDNF-left panel
GAPDH-left panel
BDNF-right panel
GAPDH-right panel

Fig 6H

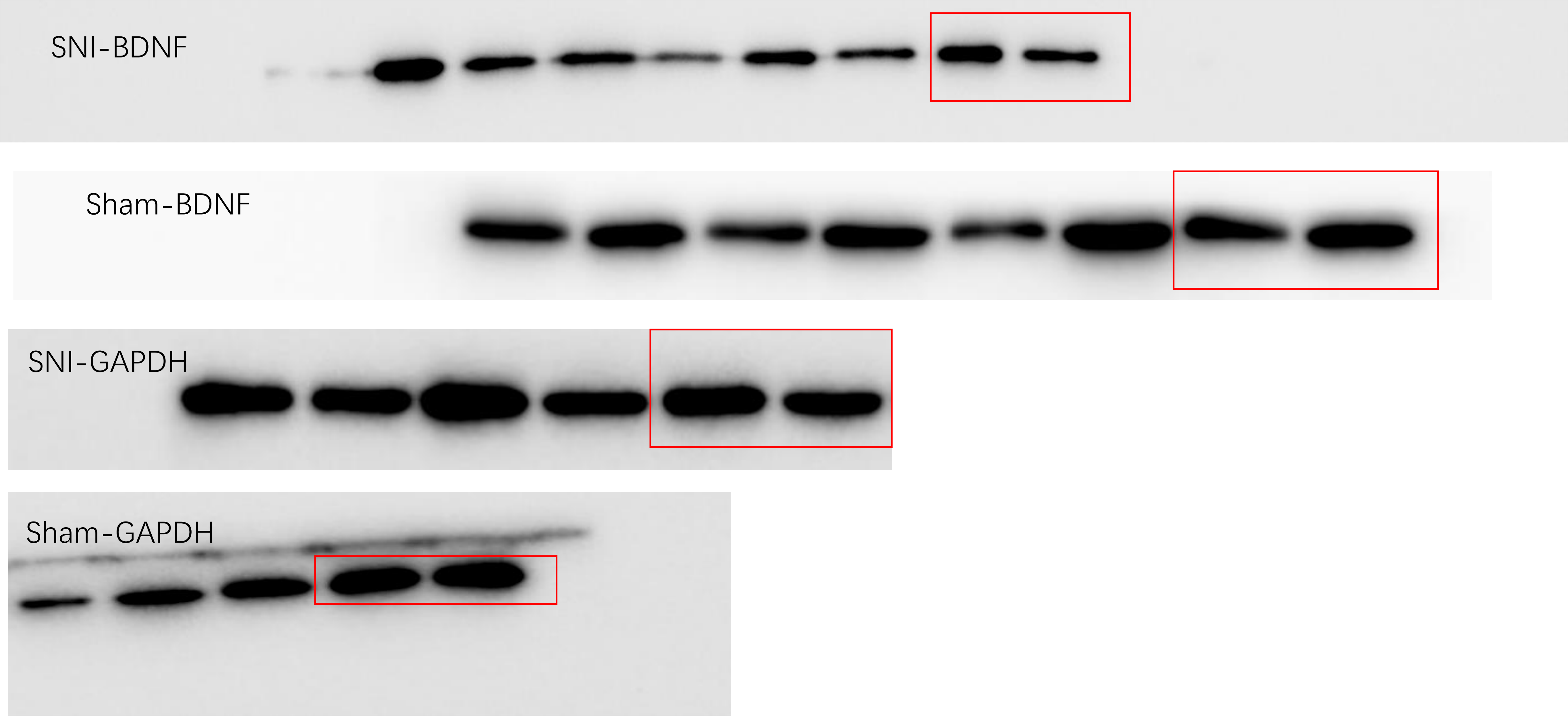

Fig 7a

BDNF-SNI rats

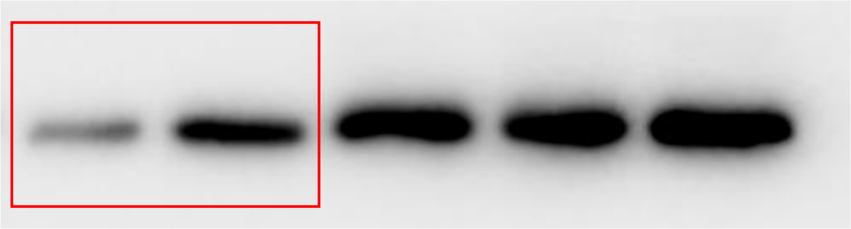

GAPDH-SNI rats

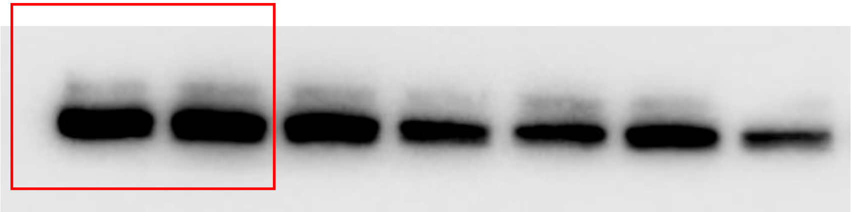

GAPDH-sham rats

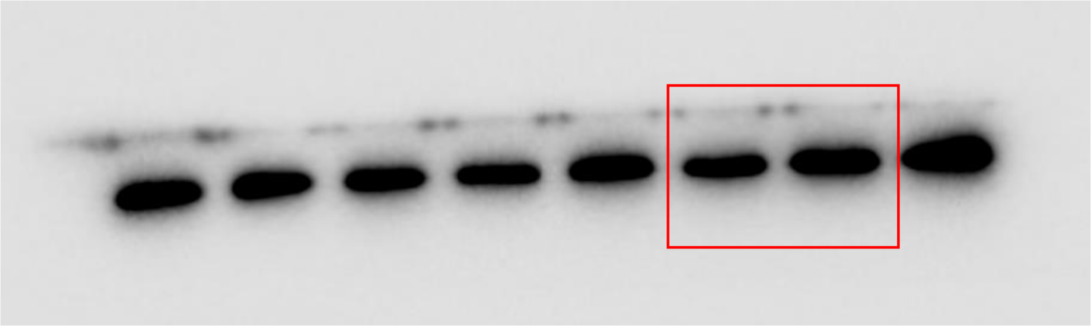

BDNF-sham rats

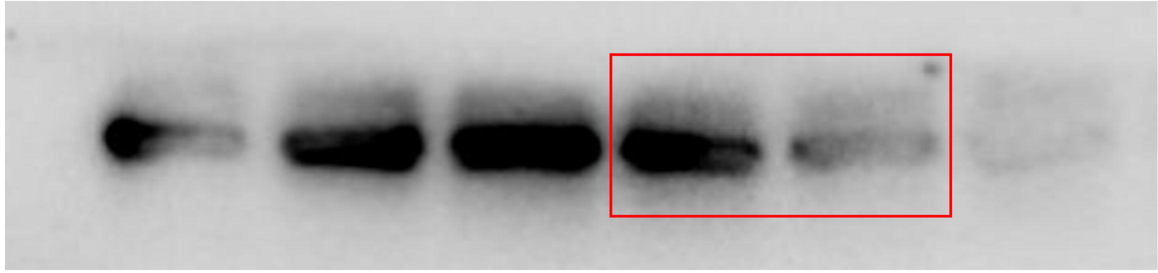

Supplement: Supplementary file 1 — Supplementary Material [file 41420_2022_880_MOESM1_ESM.pdf]
